# A systematic review of the efficacy of complementary colostrum, milk, and oral nutritional and dietary solutions on the health, growth, and gut health of suckling piglets

**DOI:** 10.1093/jas/skag095

**Published:** 2026-04-01

**Authors:** Diana Luise, Barbara Polimeni, Paolo Trevisi

**Affiliations:** Department of Agricultural and Food Sciences, University of Bologna, Viale Giuseppe Fanin, 46, Bologna, 40127, Italy; Department of Agricultural and Food Sciences, University of Bologna, Viale Giuseppe Fanin, 46, Bologna, 40127, Italy; Department of Agricultural and Food Sciences, University of Bologna, Viale Giuseppe Fanin, 46, Bologna, 40127, Italy

**Keywords:** arginine, low body weight, oligosaccharide, probiotic, sow reared

## Abstract

Pre-weaning mortality has increased with the adoption of highly prolific sow genetics. To mitigate these limitations, alternative strategies have been investigated, including supplementation with heterologous or artificial colostrum or milk (derived from different species, eg, bovine, ovine) and the use of nutritional additives, such as proteins, pre/probiotics, vitamins, and bioactive compounds to replicate the protective functions of porcine colostrum and milk. This review aims to summarize and critically evaluate the effects of early dietary interventions, including cross-species colostrum, milk replacers, and oral functional additives, on suckling piglets, focusing on survival, growth performance, and gut health-related parameters, including intestinal function, immune and oxidative status parameters, and microbial establishment. The systematic literature search was conducted using Google Scholar, PubMed, Scopus, and Web of Science, employing the search “Colostrum” AND “piglets” OR “pig”; “Milk” AND “piglets” OR “pig” until 2023. A total of 7,251 articles were identified, and 62 were retained after filtering. The selected articles were categorized into three databases: 1) Colostrum, including trials with oral colostrum interventions and swine colostrum as a control group (14 articles); 2) Milk, focusing on milk-based oral interventions with a swine colostrum or milk as control group (12 articles); and 3) Oral strategies, comprising additive-based oral interventions (eg., prebiotics, probiotics, amino acids, and combinations) with controls receiving only colostrum, milk, or water (41 articles). Although many of these alternatives partially fulfill the nutritional requirements of neonatal piglets, results across studies remain inconsistent and controversial. Milk replacers closely approximate the composition of porcine colostrum and can improve piglet growth, including low-birth-weight piglets, while also exerting positive effects on intestinal structure, immune system, and intestinal microbiota. Additional benefits may be obtained through the supplementation of other bioactive compounds (amino acids, probiotics), although the optimal dosage and timing of administration remain to be determined. To date, no substitute has proven fully capable of replicating the complex and unique functional properties of porcine colostrum. This highlights the need for continued research to develop more effective interventions that can support piglet survival and performance.

## Teaser

This review explores nutritional strategies aimed at supporting piglet survival and early growth when sow colostrum or milk is insufficient for the whole litter. It highlights the most effective interventions and identifies the practical challenges that need to be addressed to enhance their application in commercial farming.

## Introduction

Modern genetic selection for hyperprolific sows has markedly improved the productivity and economic efficiency of swine production by increasing litter size. However, this genetic progress has also introduced several biological and management challenges. Larger litters are frequently associated with prolonged farrowing duration, increased within-litter birth weight (BW) variability, and a higher proportion of low birth weight (LBW) piglets (Peltoniemi et al., 2021). These factors compromise piglet vitality and delay access to the udder, prolonging the interval between birth and first colostrum intake. Consequently, impaired early energy supply and insufficient passive immune transfer contribute to increased pre-weaning mortality, which remains a major challenge in modern pig production. Pre-weaning losses can reach 10%–20% of piglets born alive (Baxter et al. 2020), causing significant economic, environmental, and social consequences (Heuß et al. 2019). Most deaths occur within the first 1 to 2 d of life, making the periparturient period a critical window for management interventions. Among these, ensuring adequate colostrum intake is pivotal, especially for low-birth-weight piglets and in large litters, where competition for teats limits access to colostrum (Andersen et al. 2011).

Although genetic selection has increased total colostrum yield in modern sows, from approximately 5.3 to 6.9 kg (Theil et al., 2023), colostrum availability per piglet often remains insufficient. Consequently, uneven and inadequate colostrum intake restricts passive immune transfer, energy requirements for thermoregulation, and nutrients that stimulate intestinal growth and development, compromising survival, immune development, and overall health (Quesnel et al. 2012). The critical importance of colostrum in piglets is largely related to the epitheliochorial placental structure of the sow, which prevents transplacental transfer of maternal immunoglobulins. Therefore, piglets are born agammaglobulinemic and rely entirely on colostrum ingestion for immune protection (Le Dividich et al. 2005). Rich in immunoglobulins, IgG, IgA, and IgM, colostrum confers passive immunity and immediate humoral defense against pathogens (Devillers and Lessard 2007). Furthermore, mammalian colostrum is recognized as “liquid gold” for its valuable concentration of nutrients, growth factors, probiotics, prebiotics, antibodies, and other bioactive compounds, which allow the newborns to cope with the immune and energy demand in the initial extra-uterine life (Costa et al. 2023).

Despite its biological value, the limited availability of sow colostrum per piglet has prompted the development of alternative nutritional and management strategies aimed at supporting neonatal piglets, particularly those at greater risk of mortality. While various farm management practices, including manual colostrum supplementation, have been tested, these interventions can be labor-intensive and require significant time investment from farm staff. Unlike in other domestic species such as calves, the swine industry lacks organized colostrum banks, further constraining management options (Costa et al. 2023).

To overcome these limitations, several nutritional strategies have been developed to partially replicate the biological and immunological functions of sow colostrum. These include the administration of colostrum and milk from other species and/or milk replacers (MRs), as well as a variety of nutritional additives (Polo et al. 2012; Zeng et al. 2015; Berding et al. 2016). However, current formulations often differ markedly from natural sow milk in both macro- and micronutrient content and bioactive compound profiles. Bovine colostrum is the most common alternative, though interspecies differences in protein and lipid composition (Malacarne et al. 2002; Arslan et al. 2021; Nuntapaitoon 2022) and variations due to breed or parity (Nazir et al. 2020; Amatucci et al. 2022; Grodkowska et al. 2023) affect its suitability.

Bovine colostrum contains substantially higher concentrations of caseins and Igs (72 mg/mL and 76.5 mg/mL, respectively; Silva et al. 2024) than porcine milk, in which immunoglobulin levels (IgG, IgA, IgM) drop sharply within the first 24 hours after birth (12 mg/mL, Markowska-Daniel et al. 2010). Although piglets can absorb bovine Igs, these antibodies lack specificity for porcine pathogens and provide little protective benefit. For this reason, MR and supplementary milk formulations are frequently enriched with functional additives such as amino acids, proteins, vitamins, minerals, prebiotics, probiotics, and other bioactive compounds to better support piglet development (Tan et al. 2009; Reznikov et al. 2014; Dong et al. 2016; Moreira et al. 2017; Schmitt et al. 2019; Galiot et al. 2021; Luise et al. 2021b; Correa et al. 2023; Eicher et al. 2006; Ayuso et al. 2020). These interventions aim to enhance immune competence, energy supply, and overall physiological development, while also promoting the natural suckling of maternal milk. In fact, by supporting energy metabolism, immune function, and physiological resilience, both supplementary milk, MR, and functional additives used in the studies may enhance more frequent and effective access to the sow’s teats and natural suckling behavior. In addition, the continuous supplementation of MR and additives from 12-24 hours after birth throughout the entire lactation period has been shown to improve growth performance at weaning and reduce mortality rate during the suckling phase (Prims et al. 2017; Jin et al. 2020; Chem et al. 2023). Similarly, providing MR administered automatically from day 3-5 of life may further support piglets and represent a more practical alternative to artificial colostrum administration, enhancing vitality and survival (Correa et al. 2023).

In addition to survival and growth performance, early-life nutritional interventions may exert profound effects on gastrointestinal development and gut health, especially during the neonatal phase, which is recognized as a critical window for gut health imprinting in piglets (Pluske et al. 2018; Trevisi et al. 2021). According to Chalvon-Demersay et al. (2021), gut health can be defined as “the ability of the gastrointestinal system to confer resistance and resilience, enabling animals to respond and adapt to environmental and physiological challenges while maintaining optimal performance, low morbidity and mortality, and overall health.” Within this framework, gut health is structured around four interconnected pillars: 1) epithelial barrier function and nutrient absorption, 2) intestinal immune fitness, 3) oxidative stress homeostasis, and 4) microbiota balance. These domains can be evaluated through a range of measurable indicators, including intestinal morphology markers, digestive enzyme activity, immune and inflammatory biomarkers, microbial diversity and composition, and oxidative stress markers. The coordinated development of these pillars is essential for the maturation of mucosal immunity and for the maintenance of intestinal homeostasis (Pluske et al. 2018). A well-structured and balanced intestinal ecosystem contributes not only to reduced pre-weaning mortality but also to improved growth performance and the development of more resilient piglets capable of withstanding post-weaning challenges (Trevisi et al. 2021; Lee et al. 2023).

Despite numerous studies, evidence regarding the effects of early nutritional interventions, including colostrum, milk, and feed additives, on growth, gut development, and immune function remains inconsistent, highlighting the variability and complexity of these interventions (Sauer et al. 1997; Declerck et al. 2016). Data are fragmented, and a clear consensus on the optimal composition, dosage, and timing of supplementation is still lacking. Addressing these knowledge gaps is essential for the design of evidence-based, practical feeding strategies that can reliably enhance piglet survival, growth, and gut health. Therefore, the present systematic review aims to critically evaluate the effects of early dietary interventions, including cross-species colostrum and milk, MRs, and direct oral supplementation with functional additives, on suckling piglet outcomes. Specifically, this review focuses on the impact of these nutritional interventions on survival and mortality, growth performance, and gut health-related parameters, identifying current knowledge gaps and providing recommendations to guide the development of nutritional strategies to improve piglet vitality and performance.

## Material and methods

This systematic review was conducted according to the Preferred Reporting Items for Systematic Reviews and Meta-Analyses statement (Liberati et al. 2009).

### Research analysis approach

Systematic literature review was conducted in December 2023 to assess and categorize recent scientific studies on the use of colostrum, milk, MR, and oral solutions on suckling piglets using Web of Science, Scopus, PubMed, and Google Scholar databases to identify all articles written in English. To capture the widest possible range of studies, no time restriction was applied. The following keywords were used: “Colostrum” AND “piglets” OR “pig”; “Milk” AND “piglets” OR “pig”; “additive” AND “piglets” OR “pig.”

**Figure 1:**
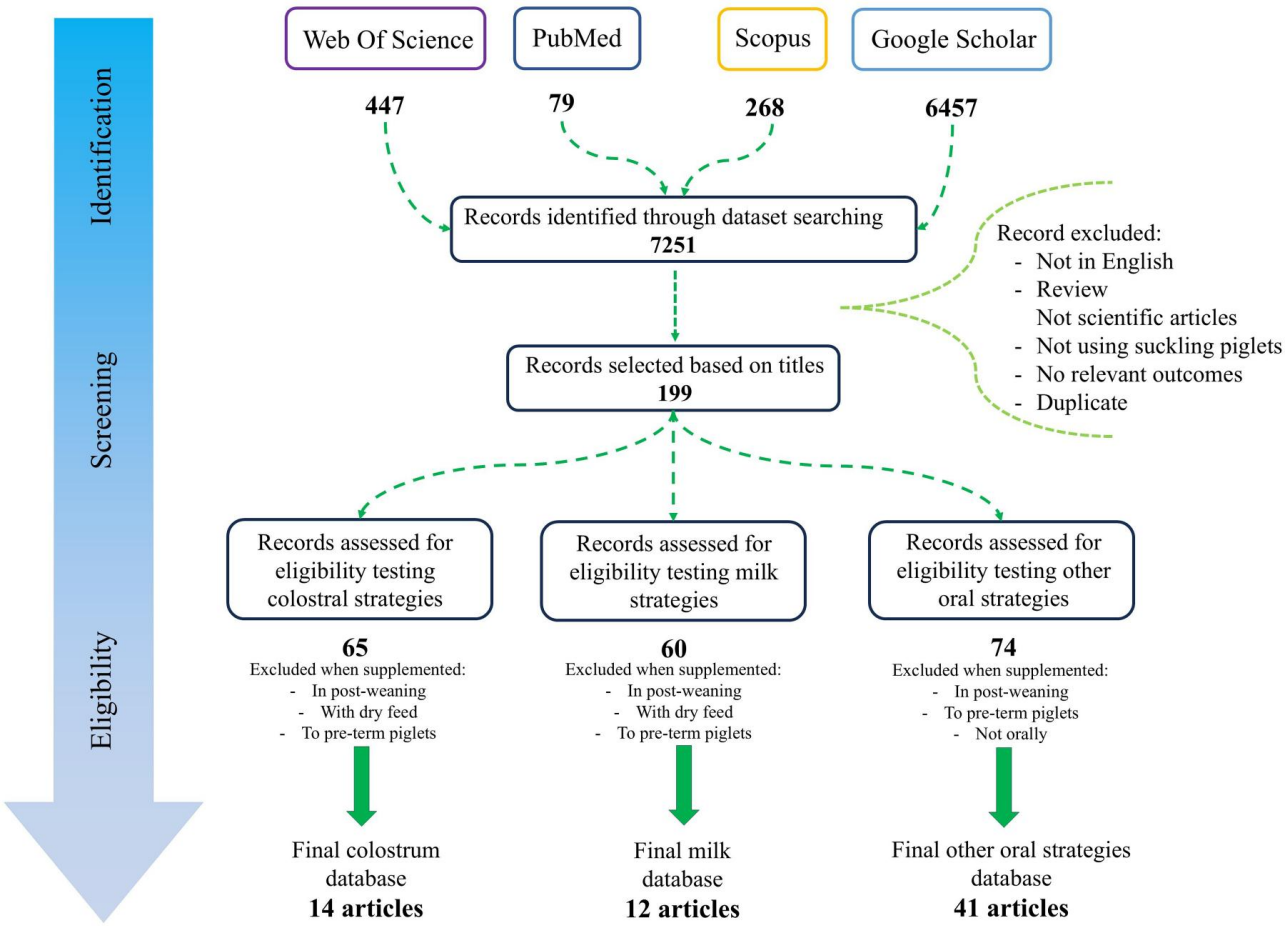
Flow diagram illustrating the selection process of studies included in the review.

This search yielded 7,251 articles. The study selection is detailed in Figure 1. Briefly, duplicate articles, articles not published in a peer-reviewed journal, articles not written in English, review letters, commentaries, conference papers, and studies not focusing on in vivo studies were initially excluded. Furthermore, articles in which the study was conducted only on sows or on post-weaning piglets were excluded. The retained studies were then divided into three different databases:

1. Colostrum database: a database focusing on oral intervention based on colostrum. For this database, the group receiving the swine colostrum (suckling piglets or colostrum-deprived piglets fed porcine colostrum artificially) was considered as control group (CO). All articles without the CO group were excluded. A total of 14 articles fulfilled the inclusion criteria for this database.
2. Milk database: a database focusing on oral intervention based on milk. For this database, the group receiving the swine colostrum and then milk was considered as a control group (CO). All articles without the CO group were excluded. 12 articles fulfilled the inclusion criteria for this database.
3. Oral strategies: a database focusing on oral intervention based on additives, including prebiotics, probiotics, amino acids (AAs), short-chain fatty acids, or a combination of different feed additives. In this case, the CO groups were represented by piglets receiving only colostrum, milk, or water. Articles in which the additive was not provided orally or was supplemented within creep feed were excluded. Forty-one articles fulfilled the inclusion criteria for this database.

### Data extraction

The effect of colostrum, milk, and oral supplement intervention was investigated on the parameters related to growth, health, and mortality and the parameters related to the four pillars of gut health described by Chalvon-Demersay et al. (2021). The four pillars are the epithelial barrier and digestion, immune fitness, the microbiota balance, and the oxidative status of the animals. In detail, the data reported in the Tables were extracted and analyzed.

For each article, the following information was identified and extracted:

- Categorization: the studies were classified based on the type of supplementation (colostrum, milk, oral strategies).
- Study design: defined the control group and the supplemented ones for each category. The dose and the timing of supplementation were also defined.
- Extraction of the results of the studies based on the gut health pillars (where investigated):
   - Growth performance and health: body weight (BW), average daily gain (ADG), gain to feed ratio (G:F), mortality and diarrhea rate, and antibiotic intervention.
   - Epithelial barrier and digestion: intestinal morphology (villous height [VH], crypt depth [CD], villous to crypt ratio [VH:CD]), intestinal integrity (tight junction proteins such as occludin, claudin, zonula occludens), intestinal activity (apparent total tract digestibility [ATTD]), and enzymatic activity (eg, lactic dehydrogenase, lactase, sucrase).
   - Immune fitness: blood parameters (eg, lymphocytes, granulocytes, glucose), Igs concentration (IgG, IgA, IgM, sIgA), and immune response indicators (eg, cytokine profile, toll-like receptor).
   - Oxidative stress homeostasis: oxidation markers (glutathione peroxidase [GPX], catalase [CAT]) and other parameters investigated in the studies.
   - Microbiota: α-diversity and the variation of abundance of certain taxa.

The percentage difference between the supplemented and control groups within each trial was calculated when the comparison was statistically significant (*P* < 0.05) or showed a tendency toward significance (*P* < 0.10). Differences were expressed as the percentage of increase or decrease (− or +) compared to the control. Additional data on colostrum/milk composition, test duration, number of piglets per group, population characteristics (including age, BW, rearing status: suckling piglets or piglets not reared with their dam, premature, LBW, or normal piglets), and type and duration of challenge were also retrieved. In addition, for the oral supplementation dataset, the type and dose of supplementation and the matrix used to deliver the supplement (milk, water) were extracted.

In the “Results and discussion” section, the results will be discussed starting from the growth performance and general health of the piglets (including diarrhea and mortality rate), followed by the intestinal health pillars and relative markers.

The full list of articles obtained from the literature search is given in Table S1 (see online supplementary material).

## Results and discussion

### Effect of the colostrum replacement

A total of 14 articles investigating the supplementation of enriched porcine colostrum, bovine colostrum, or goat colostrum in suckling piglets were retained. To enable comparison, the studies were grouped according to the type of colostrum administered: porcine vs. additional porcine (seven articles); porcine vs. bovine (seven articles); and porcine vs. goat (one article). The results are reported in Tables 1 and 2.

**Table 1:** Effect of additional doses of sows’ colostrum on gut health and growth parameters of suckling piglets.

Origin of colostrum	Control group	Group	Dose and timing	Growth performance and health	Epithelial barrier and digestion	Immune fitness	Reference
**Porcine**	Sow-reared piglets	Sow-reared piglets + colostrum	30 mL in 24 h	↑ weaning weight homogeneity, ADG; ↓ mortality, diarrhea, antibiotic intervention	–	–	Miguel et al. (2021)
		Sow-reared piglets × 24 h + colostrum	50 mL in 4 h	= BW, ADG, mortality	–	–	Viott et al. (2018)
		Sow-reared piglets × 24 h + energy supplement	8 mL in 4 h	= BW, mortality ↑ ADG			
		Sow-reared piglets × 24 h + colostrum + energy supplement	50 mL + 8 mL in 4 h	= BW, ADG, mortality			
		Sow-reared piglets (LBW) + colostrum	15 mL in 12h	= BW, ADG, mortality	–	↑ IgG	Muns et al. (2014)
		Colostrum-deprived piglets + colostrum + energy supplement	120 mL colostrum + 4 mL in 24 h	↓ ADG (24h), = weaning BW, ADG, mortality	–	= IgG	Moreira et al. (2017)
		Colostrum-deprived piglets + colostrum + energy supplement	200 mL colostrum + 4 ml in 24 h	= ADG (24 h), weaning BW, ADG, mortality		↑ IgG	
		Colostrum-deprived piglets + 120 mL of colostrum	120 mL in 24 h	↓ ADG (24h), = weaning BW, ADG, mortality		= IgG	
		Colostrum-deprived piglets + 200 mL of colostrum	200 mL in 24 h	= weaning BW, ADG, mortality		↑ IgG	
		Colostrum-deprived piglets + colostrum	220 mL/kg BW in 24 h	–	= VH, CD (duo, jej, ile)	–	Mei et al. (2006)
		Colostrum-deprived piglets + colostrum	20 mL/3 h in 12 h	↓ ADG (0–12 h) = weaning BW, ADG, mortality	–	↓ porcine IgG (12 h – 10 d)	Martínez Miró et al. (2020)
	Colostrum-deprived piglets + 20 mL of whole sow colostrum	Colostrum-deprived piglet + defatted colostrum	17.8 mL/2 h in 24 h	–	↑ protein (intestinal mucosa) ↓ FABP (intestinal mucosa)	–	Reinhart et al. (1992)

Abbreviations: ADG, average daily gain; BW, body weight; CD, crypt depth; Duo, duodenum; Ig, immunoglobulin; Ile, ileum; Jej, jejunum; VH, villous height.

* colostrum-deprived piglet = Piglets that were separated from the sow before colostrum intake.

**Table 2:** Effect of bovine and ovine colostrum on gut health and growth parameters of suckling piglets.

Origin of colostrum	Control group	Group	Dose and timing	Growth performance and health	Epithelial barrier and digestion	Immune fitness	Reference
**Bovine**	Sow-reared piglets	Sow-reared piglets (LBW) + colostrum replacer	10 mL in 12 h	= BW, ADG, mortality	–	↑ IgG, IGF-1	Muns et al. (2017)
		Sow-reared piglets + colostrum preparation	1 mL/d × 1–3 d of life	= BW, ADG, mortality, diarrhea	–	↓ total protein blood, β-globulin	Viehmann et al. (2015)
		Colostrum-deprived piglets + colostrum	Ad libitum × 3 d of life	–	–	= C-CH50 (48–72 h) ↑ A-CH50 (24, 48 h) = IgG	Renshaw and Gilmore (1980)
		Colostrum-deprived piglets + colostrum			= VH (duo, jej), ↑ VH (ile), = CD (duo, jej, ile)	–	
		Bovine colostrum*	20 mL	= weaning BW, diarrhea ↓ mortality	–	–	Bandyk and Hines (1986)
		Sow-reared piglets + colostrum	4 g/d from d 5 to d 10 of life	–	–	↑ CD3^−^ CD16^+^ leukocytes ↓ *IL5, IL15* (jej)	Lo Verso et al. (2020)
		Sow-reared piglets + colostrum + Vit. A + Vit. D + Cu	4 g/d from d 5 to d10 of life			↑*TNF*, *osteopontin* (intestine)	
		Colostrum-deprived piglets + colostrum	10 mL/kg BW/2h in 12 h	–	–	↓ porcine IgG	Goncharova et al. (2017)
**Infant formula**		Colostrum-deprived piglets + infant formula	10 mL/kg BW/2h in 12 h				
**Goat**		Colostrum-deprived piglets + colostrum	20 mL/3h in 12 h	↓ ADG (0–12 h) = weaning BW, ADG, mortality	–	↓ porcine IgG (12 h – 10 d)	Martínez Miró et al. (2020)

Abbreviations: A-CH50, alternative complement pathway activity; ADG, average daily gain; BW, body weight; C-CH50, classical complement pathway activity; CD, crypt depth; CD3^−,^ cluster of differentiation 3; CD16^+^, cluster of differentiation 16; Duo, duodenum; Ig, immunoglobulin; Ile, ileum; IGF-1, insulin growth factor-1; Jej, jejunum; TNF, tumor necrosis factor; VH, villous height.

* Not specified if sow-reared or colostrum-deprived.

Studies on porcine colostrum supplementation have shown that providing an additional dose of colostrum does not always reduce mortality rates, as demonstrated by Viott et al. (2018) and Muns et al. (2014). However, the amount of colostrum consumed plays a crucial role; for instance, in colostrum-deprived piglets, 120 mL of colostrum administered during the first 12 h of life (enriched with whey proteins, refined coconut oil, L-carnitine, butyric acid, nucleotides, chromium picolinate, and vitamin E) was not enough to maintain the same survival rate as naturally suckling piglets (Moreira et al. 2017), whereas giving 200 mL of porcine colostrum achieved comparable survival. According to the study by Miguel et al. (2021), administering an additional dose of porcine colostrum (30 mL in 12 h) reduced the mortality rate by 60.1%, as well as the diarrhea rate (by -39.6%) in LBW piglets. Similarly, the additional availability of porcine colostrum was also found to maintain (Muns et al. 2014) or improve the piglets’ ADG in the LBW piglets (26.6%, Miguel et al. 2021), making them able to be competitive in the litter. This effect may be attributed to greater intake of maternal colostrum and milk following the initial artificial assistance with colostrum supplementation. In fact, colostrum is known to be a key source of nutrition and immunity for piglets in the first 24 h post-farrowing. Porcine colostrum is particularly rich in proteins, lipids, and carbohydrates (mainly lactose), the latter being an essential source of energy for thermoregulation in piglets (Inoue and Tsukahara 2021). On average, a piglet requires 65.7 kcal/kg for maintenance in the first 24 h (Le Dividich et al. 1994), the additional energy being expended for growth. The protein component of swine colostrum consists of α, β, and κ caseins, albumin, α-lactoglobulin, β-lactalbumin, and immunoglobulins, all of which are important for neonatal development (Devillers and Lessard 2007). Immunoglobulins, especially IgG, are essential antibodies for the piglet’s immune system, ensuring a defensive barrier. Le Dividich et al. (2005) showed that piglets with blood IgG levels of around 40 mg/mL were stronger and healthier. According to the literature, a minimum of 200 mL within the first 24 h post-farrowing is required to increase the piglet survival (Devillers et al. 2004; Ferrari et al. 2014) and the piglets’ blood IgG levels by 20.5%–25.5% (23.1 vs. 29 mg/mL) (Moreira et al. 2017). These data were confirmed by Moreira et al. (2017) and Martínez Miró et al. (2020), who demonstrated that 100–120 mL of colostrum was insufficient to enhance IgG concentration, growth, or survival rates in colostrum-deprived piglets within the first 24 h of life. On the contrary, the study by Muns et al. (2014) found that LBW piglets separated from the sow for only the first 4 h of life and supplemented with 15 mL of porcine colostrum within increased IgG concentration 4 d after birth. However, it should be noted that these quantitative estimates in these latter studies derive primarily from experimental models involving piglets that were completely or temporarily separated from the sow and therefore do not allow a precise determination of the colostrum intake required under natural suckling conditions.

The epithelial barrier, oxidative status, and microbiota pillars were poorly studied. Only the study by Reinhart et al. (1992) showed that piglets receiving approximately 20 mL of porcine colostrum within the first 24 h of life showed an increase in intestinal proteins in the ileum compared with the group fed a lactose/electrolyte solution. This increase in protein may have resulted from the stimulation of the intestinal mucosa by colostrum proteins. In contrast, in colostrum-deprived piglets, the administration of 220 mL/kg BW during the first 24 h of life appeared to be inadequate to induce any alterations in gut morphology (Mei et al. 2006).

Despite the positive effects associated with the use of supplemental porcine colostrum in newborn piglets, especially to LBW piglets, it is well known that this strategy is difficult to implement in practice. The use of colostrum from other dairy species appears to be a more feasible and practical alternative. Therefore, several studies have investigated the effects of bovine colostrum supplementation in newborn piglets.

The collected articles on the use of bovine colostrum indicated that its implementation to piglets that were allowed to suckle from the sow did not lead to an increase in BW or ADG during suckling (Viehmann et al. 2015) and at weaning (Bandyk and Hines 1986). Regarding the mortality rate, no difference (12.8% in suckling piglets vs 10.7% in suckling piglets + bovine colostrum) was observed by Viehmann et al. (2015) at day 10 of life, following a supplementation with 1 mL/d from d 1 to 3 of life. However, according to Bandyk and Hines (1986), the administration of 20 mL of bovine colostrum within the first 12 h of life reduces the total mortality rate by 7.7% in newborns with BW > 1.35 kg and by 65.6% in newborns with BW <1.35 kg. These results suggest that bovine colostrum can contribute to increasing the survival and robustness of piglets. The positive effect may be attributed to the additional nutrients or bioactive compounds present in the bovine colostrum. Unlike porcine colostrum, supplementing the piglets (separated from the sow) only with bovine colostrum did not allow for the transfer of specific porcine IgG; in fact, porcine IgG in piglets’ blood had a reduction ranging from 74% (Jensen et al. 2001) to 99% (Goncharova et al. 2017). Nevertheless, when comparing piglets fed bovine colostrum with those that received only maternal colostrum, bovine colostrum appeared to stimulate immune maturation. As observed by Lo Verso et al. (2020), the bovine colostrum led to a higher blood expression of cytotoxic T cells and CD3^-^ and CD16^+^ leukocytes and a lower expression of Interleukin-15 (*IL15)* and *IL5* before (d16) and after weaning stress (defined as the combined nutritional, social, and environmental challenges associated with separation from the sow, transition to solid feed, and increased inflammation and susceptibility to disease). The effect could be associated with the high quantity of bovine lactoferrin (bLF), which is known to stimulate natural killer cells, such as CD3 and CD16 (Kuhara et al., 2006). Furthermore, these cells are known to gradually increase with the maturation of the immune system, especially after weaning (Talker et al. 2013), and to interact with cytokines (Vivier et al. 2011). This interaction could explain the lower expression of *IL15* and *IL5*, which play a key role in modulating both innate and adaptive immune cell homeostasis and the inflammatory process (Lo Verso et al. 2020).

Regarding the effect of bovine colostrum on the gut integrity pillar, only one study was found. At the intestinal level, villus height and crypt depth in the duodenum, jejunum, and ileum at d 1 post-farrowing were investigated in the study by Mei et al. (2006). The study found no difference in the morphological parameters (duodenum and jejunum) between piglets reared with the dam and piglets separated from the dam artificially fed bovine colostrum (220 mL/kg BW in 24 h). However, villus height in the ileum was increased in the supplemented piglets.

Only one article was identified regarding the use of goat colostrum. Since goat colostrum is more digestible than bovine colostrum (Turkmen 2017) and has a high nutritional value, the authors hypothesized that it could be a potential alternative for newborn piglets. Martínez Miró et al. (2020) reported that replacing porcine colostrum with 100 mL of goat colostrum within the first 12 h reduced ADG at 12 h (−11.6%), likely due to lower total colostrum intake in artificially reared piglets. However, BW at weaning, mortality, and diarrhea incidence were unaffected. Although serum porcine IgG levels were initially reduced, the absorption efficiency of goat IgG was comparable to that of porcine IgG, indicating that piglets can absorb non-species-specific immunoglobulins, even though goat-derived Igs will not have the same immune defense functionality as species-specific ones.

Overall, supplemental porcine colostrum remains the most effective strategy for improving early survival, growth, and immune development, particularly in LBW piglets. However, practical limitations often restrict its availability, making bovine colostrum a viable alternative despite the lack of species-specific IgG transfer, and goat colostrum represents a promising but still insufficiently investigated option. Due to the heterogeneity of experimental designs evaluating colostrum from different species, defining optimal supplementation dosages remains speculative. Nevertheless, the limited available evidence suggests that goat IgG is absorbed more efficiently than bovine IgG, indicating that piglets can absorb this kind of non-species-specific immunoglobulins. Despite this improved absorption, goat colostrum does not provide an adequate level of passive immunization to fully replace sow colostrum.

### Effect of milk replacement

A total of 12 articles investigating the supplementation of MR formula (powder or liquid) to suckling piglets were identified. Seven out of 12 articles investigated the effect of MR during early life (1–10 d of life), while the remaining five articles studied the effect of MR in the first 2–3 wks of piglets’ life (14–21/23 d). The effects of the MRs on the performance and health, and on the four pillars of gut health, are summarized in Table 3.

**Table 3:** Effect of milk replacer supplementation on gut health and growth parameters of suckling piglets.

Origin of milk	Control group	Group	Dose and timing	Growth performance and health	Epithelial barrier and digestion	Immune fitness	Microbiota	Reference
**Milk replacer^1^**	Sow-reared piglets	Sow-reared piglets + milk replacer	Ad libitum at d 7 of life	↑ Weaning BW, ADG ↓ Diarrhea	↑ OCLN, CLD, ZO-1, TRL-4	↓ TNF-α, IL-8	↓ *Lactobacillus, Firmicutes, Bacteroidetes, Proteobacteria*	Jin et al. (2020)
		Sow-reared piglets + milk replacer	5–15 mL x 24–36h of life	= Weaning BW, ADG, mortality	–	–	–	Van Tichelen et al. (2023)
		Sow-reared piglets + milk replacer (powder)	Ad libitum till weaning	↑ Weaning BW, ADG ↓ Mortality	–	–	–	Chem et al. (2023)
		Sow-reared piglets + liquid milk formula (feeding trough)						
		Sow-reared piglets + liquid milk formula (feeding bucket)		= weaning BW, ADG, Mortality				
		Sow-reared piglets + milk replacer	Ad libitum till weaning	↑ weaning BW, ADG	–	–	–	Wolter et al. (2002)
		Sow reared piglets + milk replacer (MS1) administered automatically	Ad libitum × 14 d	= weaning BW, ADG ↓ Mortality	–	–	↑ α-diversity, Lachnospiraceae	Correa et al. (2023)
		Sow reared piglets + milk replacer (MS1) administered manually		–			↑ α-diversity, Oscillospiraceae	
		Sow reared piglets + milk replacer (MS2) administered manually					↑ α-diversity	
		Milk replacer*	20 mL	= Weaning BW, Diarrhea, Mortality	–	–	–	Bandyk and Hines (1986)
		Colostrum-deprived piglets + milk replacer	Ad libitum × 19 d	↑ Weaning BW	–	↓ M cell densities (ile)	–	Prims et al. (2017)
		Colostrum-deprived piglets + infant formula	221 mL/kg BW in 24 h	–	= VH, CD (jej), ↓ VH, CD (ile)		–	Mei et al. (2006)
		Sow-reared LBW piglets + Milk replacer	2.5 mL × 7 d	= BW, Mortality	–	= IgG, IGF-1	–	Van Tichelen et al. (2021)
**Bovine**		Milk-deprived piglets + milk formula with colostral whey	Ad libitum x 10 d	↓ BW (d 10)	↓ VH ↑ CD, OCLN ↓ Mannitol ↓ Lactase = Sucrase, Maltase, Aminopeptidase, ↓ Dipeptidyl peptidase	–	–	De Vos et al. (2014)
		Sow-reared piglets + skim milk formula	Ad libitum × 20 d	↑ BW, ADG (d 10–d 20)		–	–	Dunshea et al. (1999)
**Porcine**		Colostrum-deprived piglets + mature milk	220 mL/kg BW in 24 h	–	↑ VH (jej), ↓ VH (ile), ↓ CD (jej, ile), = V:C (jej)	–	–	Mei et al. (2006)
	Colostrum-deprived piglets + 20 mL of sow colostrum	Colostrum-deprived piglets + 20 mL of defatted sow milk	17.7 mL/2h × 24h	–	↓ Protein (intestinal mucosa), FABP mucosa, soluble protein (intestinal mucosa)	–	–	Reinhart et al. (1992)
	Sow-reared piglets	Colostrum-deprived piglets + sow milk replacer formula	360 mL/kg BW/d × 21 d	–	–	↓ NK cells ↓ IL-2	–	Liu et al. (2013)
		Colostrum-deprived piglets + sow milk replacer formula + bovine lactoferrin	360 mL/kg BW/d + 1g/L × 21 d			= NK cells ↓ IL-2		

Abbreviations: ADG, average daily gain; BW, body weight; CD, crypt depth; CLD, claudin, Ig, immunoglobulin; Ile, ileum; IGF-1, insulin growth factor-1; IL, interleukin; Jej, jejunum; NK, natural killer cells; OCLN, occluding; TLR, toll-like receptor; TNF, tumor necrosis factor; VH, villous height; V:C, villous to crypt ratio; ZO-1, zonula occludens.

1 Origin of milk not known.

* Not specified if sow-reared or colostrum-deprived.

From the data analysis, the ad libitum MR supplementation to piglets (allowed to suckle from the sow) improved the BW at weaning with a percentage ranging from 15.9% (Wolter et al. 2002) to 36.4% (Prims et al. 2017), while De Vos et al. (2014) reported a reduction in the BW at 10 d of life. Furthermore, Dunshea et al. (1999) observed a sex-dependent effect of the MR; in both sexes, BW at weaning (d 20) increased, but in females, BW was significantly higher: by 15.6% compared with the control group (6.94 kg vs 6.00 kg). When comparing male and female piglets in the treatment groups, females had a 10% higher BW than intact males (6.94 kg vs 6.53 kg), indicating that females grew faster. This sexual dimorphism may be related to differences in digestive function between young males and females. Overall, MR supplementation positively influenced BW and ADG during d 10–20 of lactation (Dunshea et al. 1999). Chem et al. (2023) compared different litter feeding techniques, administering MR ad libitum (until weaning) either via trough or bucket. The piglets fed by trough showed a significant increase in the BW compared to piglets fed by bucket (9.28 kg vs 7.52 kg). Consuming MR from a trough was easier for the piglets; indeed, they ingested both MR and sow milk, whereas piglets fed from buckets preferred sow milk, likely because the bucket was more difficult for them to access. No differences in weaning BW, ADG, diarrhea incidence, or mortality rate were observed when 20 mL of MR was supplemented to sow-reared piglets. Examining ADG, the collection of studies showed a similar trend to BW, with increases at weaning ranging from 9.2% (Correa et al. 2023) to 57.7% (Prims et al. 2017) in piglets supplemented with MR (ad libitum for 14–19 d) compared to the control group. Milk supplementation enhanced the daily growth rate of the piglets and consequently their performance at weaning. Piglets receiving MR ingested more milk than those fed solely on sow milk and therefore were able to allocate more energy toward growth. This positive effect may also be related to an increase in insulin-like growth factor 1 (IGF-1). Indeed, MR supplementation increased blood IGF-1 concentrations by 36.1% compared with non-supplemented piglets (27.46 ng/mL vs. 20.17 ng/mL, Van Tichelen et al. 2021). High concentrations of IGF-1 are markers for regular bone and tissue growth for normal development. Dauncey et al. (1994) showed that the absence of prenatal nutrition significantly affects the levels of IGF (4.2 nmol/L vs 1.4 nmol/L), resulting in an impact on the growth of piglets.

In addition to the improvement in the growth performance, the addition of MR to the piglets’ diets improved their survival when supplemented ad libitum (Chem et al. 2023; Correa et al. 2023). Van Tichelen et al. (2023) discriminated the piglets into very low BW (VLBW: < 0.750 kg) and LBW (0.750–1.0 kg) piglets and showed that feeding them with a supplemental dose of MR equal to 1 × 5 mL on d 1 or 1 × 5 mL on d 1 + 2 × 5 mL on d 2 of life (three doses in 36 h) did not strongly modify their mortality rate. In fact, the latter was primarily influenced by piglet BW and by the perinatal management rather than by the MR supplementation. The same outcomes were obtained in the past by Bandyk and Hines (1986), who found no significant differences in mortality rate following a 20 mL MR supplementation across piglets of different weight categories (small: <1.04 kg; medium: 1.04–1.32 kg; large: >1.36 kg).

Only one of the selected studies examined the effect of MR on diarrhea incidence. Jin et al. (2020) reported that administering MR ad libitum, six times daily for 10 mins per feeding, from d 7 of life until weaning, reduced litter diarrhea incidence by 39.7%.

Considering the immune fitness pillar, blood IgG concentration was the most investigated parameter. Plasma IgG levels were 94.2% lower in piglets (colostrum-deprived) receiving MR during the first days of lactation, particularly on d 2 (52.2 g/L vs 3 g/L) (Jensen et al. 2001). In contrast, at weaning, IgG concentration was higher in the MR group than the control group (5.41 g/L vs 2.55 g/L), especially in LBW piglets (Van Tichelen et al. 2021). Regarding inflammatory biomarkers, a significant decrease of 80% in Tumor Necrosis Factor α (TNFα), 75% in IL-8, and an increased level of the Toll-Like Receptor (TLR-4) by 50% were observed, followed by MR supplementation in the study by Jin et al. (2020). A decrease in Natural Killer (NK) cells and IL-2 was observed in colostrum-deprived piglets fed with porcine MR during lactation by Liu et al. (2013). These results suggested that early nutritional interventions, such as MR supplementation or the provision of porcine milk formula, can modulate the inflammatory response and immune cell populations in neonatal piglets, potentially influencing their susceptibility to infections and overall immune development.

Observing the epithelial barrier and digestive pillar, indicators such as villus height and crypt depth have been mainly investigated in the studies. The use of MR, as an alternative to the maternal colostrum, decreased the villus height by 14.5% (De Vos et al. 2014) (on d 10 of lactation), while the crypt depth had an increase of 52.7% (on d 10 of supplementation, De Vos et al. 2014) compared with the piglets of the control group. Contrasting results have been obtained by Mei et al. (2006), with a reduction in villous height and crypt depth in the ileum of piglets fed 220 mL/kg BW of infant formula in the first 24 h of life. The shorter villus height reduced the intestinal absorption and intestinal enzymatic activity, especially of lactase, by 57% (De Vos et al. 2014). Jin et al. (2020) showed the effects of early administration (MR ad libitum for 7 d) in the small intestine of piglets weaned at 21 d. The results showed a significant increase in levels of occludin by 30%, zonula occludens-1 (ZO-1) by 30%, and claudin-1 (CLDN) by 350% in the barrier proteins. In contrast, Reinhart et al. (1992) reported a decrease in intestinal mucosal protein and fatty acid-binding protein (FABP) levels in piglets fed defatted porcine colostrum compared to the whole colostrum group, suggesting that the lipid fraction of whole colostrum plays a key role in inducing intestinal FABP activity.

Supplemented piglets with MR had controversial effects on gut microbiota; Correa et al. (2023) obtained an enhanced α-diversity and an increase in some short-chain fatty acid (SCFAs)-producing bacteria such as Lachnospiraceae and Oscillospiraceae by supplementing MR to piglets for 14 d during suckling; on the contrary, Jin et al. (2020) observed a decrease in microbial diversity and beneficial bacteria such as *Lactobacillus* and *Romboutsia*. This reduction in potentially beneficial bacteria did not compromise the intestinal health of the animals, as MR supplementation was associated with a decreased incidence of diarrhea (Jin et al. 2020).

Overall, the reviewed studies indicate that MR supplementation during the suckling period can enhance growth performance, increase IGF-1 levels, and, in some cases, reduce mortality and diarrhea incidence. Early MR feeding may also modulate immune responses by influencing immunoglobulin concentrations, inflammatory biomarkers, and immune cell populations, potentially improving disease resilience. Although effects on gut morphology and microbiota composition were inconsistent across studies, with both positive and negative shifts in villus structure and bacterial communities, these changes did not necessarily impair intestinal health. Collectively, the evidence supports MR supplementation, particularly when provided alongside sow milk (for about 2 wks during lactation), as a viable nutritional strategy to improve neonatal piglet performance and health. However, variability in outcomes highlights the need for further research to optimize formulation, timing, and delivery methods.

### Effect of direct oral nutritional and feeding supplements on suckling piglets

Direct oral nutritional and feeding supplements to MR have gained attention in pig nutrition as potential strategies to support piglets’ health during the suckling period. The provision of these supplements, administered within the initial days of life or during the lactation period, may encompass AAs and proteins, energy sources, vitamins, probiotics, prebiotics, and other bioactive compounds to support gut health and growth performance.

### Effect of amino acids and protein supplementation

AAs, the building blocks of proteins, are also essential for various physiological functions, including tissue development, enzyme production, and immune responses. In piglets, the intake of essential AAs such as lysine, methionine, threonine, and valine are of paramount importance for optimal growth and health (Chalvon-Demersay et al. 2021).

It is evident that the AAs composition of sow colostrum and milk exerts a substantial influence on the development of the intestinal tract, immune system, and overall growth and survivability of the piglet  (Luise et al., 2021a). A lack of specific AAs has been demonstrated to result in diminished growth rates and elevated vulnerability to diseases (Chalvon-Demersay et al. 2021). It is therefore necessary to supplement certain of these compounds in the diet of suckling piglets.

In total, eight studies investigated the effects of the oral supplementation of AAs; in particular, arginine (Arg) (four studies), N-carbamylglutamate (NCG), a derivative of glutamate (two studies), and glutamine (Gln) (one study) were tested in vivo. The results of the studies are reported in Table 4.

**Table 4:** Effects of amino acids on gut health and growth parameters of suckling piglets.

Additive	Control Group	Group	Dose and timing	Growth performance and health	Epithelial barrier and digestion	Immune fitness	Oxidative stress homeostasis	Reference
**Arginine**	Milk-deprived piglets fed milk-based formula	Milk-deprived piglets + milk-formula 0.2% (Arg)	0.2% (Arg) × 21 d	= Weaning BW, ADG	–	= WBC, ↓ Lymphocytes, ↑Granulocytes, ↑IgG, TNF-α, ↓ IL-8, = IgM, IL-1β	–	(Tan et al. 2009)
		Milk-deprived piglets + milk-formula 0.4% (Arg)	0.4% (Arg) × 21 d	↑ Weaning BW, ADG		↓ WBC, Lymphocytes, ↑Granulocytes, ↑IgG, IgM, IL-1β, = IL-8, TNF-α		
		Milk-deprived piglets + milk-formula 0.6% (Arg)	0.6% (Arg) × 21 d			↓ WBC, Lymphocytes, = Granulocytes, ↑IgG, IgM, IL-1β, = IL-8, TNF-α		
		Milk-deprived piglets + milk-formula 0.8% (Arg)	0.8% (Arg) × 21 d			= WBC, Lymphocytes, Granulocytes, ↑IgG, IgM, IL-1β, ↓ IL-8, = TNF-α		
	Milk-deprived piglets fed milk replacer	Milk-deprived piglets + milk replacer + Arg	0.5% × 21 d	↑ Weaning BW, ADG, G:F	–	–	–	Zheng et al. (2018)
			1% × 21 d		↑ VH (jej, ile) ↑ CLD-1 (jej)	↓ IL-1, TNF-α (jej) ↑ insulin, ornithine = lysine, leucine, isoleucine, valine, phenylalanine	↑ *GPX* = GPX, CAT (jej)	
			1.5% × 21 d	= Weaning BW, ADG ↑ G:F	–	–	–	
	Milk-deprived piglets fed sterile saline (0.9%)	Milk-deprived piglets + 250 mg/kg BW x 13 d	250 mg/kg BW × 13 d	↑ Weaning BW, ADG	↑ VH, VH:CD (jej, ile), ↑ villus surface area (ile) ↑ lactic dehydrogenase (ile, jej)	*↓ IL-1β*, *IFN-γ, IL-2 ↑*IgA, IgG, sIgA	↑ NO, NOS (serum, ileum)	Dong et al. (2022)
	Milk-deprived piglets fed milk replacer	Milk-deprived piglets fed milk replacer + L-Arg	1.08 g L-Arg/kg BW × 21 d	= Weaning BW, ADG	= ATTD (GE, CP)	= glucose, urea, ↑ NEFA	–	Madsen et al. (2018)
**Carnitine + Alanine**		7-day-old-pig fed milk replacer + L-carnitine + L-ala	0.40 g L-carnitine + 5.11% L-alanine/kg BW × 21 d		↓ ATTD (GE, CP)	= glucose, NEFA, ↑ urea		
**N-carbamylglutamate**	1-d-old piglets (sow-reared) + 0.52 g/kg BW (Ala)	1-d-old piglets (sow-reared) + Ala + NCG (50 mg)	50 mg/kg BW NCG × 14 d	= BW, ↑ADG (d1–d14)	↑ lactase (jej) (d7)	↑IgA (d14), = IL-6, IL-1, sIgA (ile)	–	Zeng et al. (2015)
		1-d-old piglets (sow-reared) + Ala + NCG (100 mg)	100 mg/kg BW NCG × 14 d	= BW, ADG	= lactase (jej) (d7)	–		
	Sow-reared piglets	Sow-reared piglets + NCG	50 mg/kg BW of NCG every 12 h for 7 d	↑ BW	–	–	–	Frank et al. (2007)
**Glutamine**	Sow reared piglets + 1.22 g/kg BW/d (Ala)	Sow-reared piglets + Gln	1 g/kg BW/d (Gln) × 12 d	–	= VH, VW, CD, VH:CD	–	–	Schregel et al. (2022)

Abbreviations: ADG, average daily gain; ATTD, apparent total tract digestibility; BW, body weight; CD, crypt depth; CLD, claudin; CP, crude protein; Ig, immunoglobulin; GE, gross energy; G:F, gain to feed ratio; GPX, glutathione peroxidase; Ile, ileum; IGF-1, insulin growth factor-1; IL, interleukin; Jej, jejunum; NEFA, non-esterified fatty acids; NO, nitric oxide; NOS, nitric oxide synthase; sIg, secretory immunoglobulin; TNF, tumor necrosis factor; VH, villous height; VH:CD, villous height to crypt depth ratio; VW, villous width; WBC, white blood cell.

Arginine is considered a functional AA for piglets as it is involved in a wide number of metabolic pathways (Wu et al. 2007; Ji et al. 2019), and it was recently highlighted as a pivotal AA to improve gut health in immediate post-weaning piglets (Chalvon-Demersay et al. 2021; Luise et al. 2023). Nevertheless, Arg plays a pivotal role for piglets; its concentration in sows’ colostrum and milk are quite inadequate. Even when directly supplemented to sows’ diets, Arg is quickly metabolized into proline, ornithine, and nitric oxide (NO) (Virdis et al. 2024), limiting the Arg transfer to piglets; therefore, a possible solution is to supplement Arg directly to suckling piglets.

According to Dong et al. (2022), the supplementation of 40 mL of Arg (250 mg per kg of BW/piglet for 13 d) allowed for an increase in the BW during suckling by 5% and reduced the loss of ADG in the first 3 d post-weaning by 45%. In the same way, Madsen et al. (2018), who administered 1.08 g/kg BW per day of L-Arg in a MR formula, Zheng et al. (2018), who supplemented MR with different percentages (0.5%, 1.0%, or 1.5%) of L-Arg and Tan et al. (2009), who supplemented MR with 0.2% until weaning, obtained comparable results, demonstrating the positive effects of Arg in supporting piglet growth performance. The study by Zheng et al. (2018) reported an enhanced ADG of LBW piglets supplemented with 0.5% and 1.0% L-Arg by 13.6% and 18.2%, respectively, suggesting that the better Arg dose to sustain piglets’ ADG was 1.0% in the case of LBW piglets compared to the negative control; the authors also observed an increase in blood Arg by 20%. Comparable results were reported by Tan et al. (2009), who supplemented a milk-based diet with different doses of Arg (0.2%–0.8% of Arg × 21 d). Piglets receiving 0.6% or 0.8% Arg showed increased BW and ADG during both the first and second weeks of treatment compared to the control group. Notably, the 0.4% Arg supplementation led to better growth performance than the control piglets (8%) at d 14 of life.

Arg is also recognized to have a positive effect on gut health parameters in older pigs. In fact, Zhu et al. (2013) observed that the supplementation of Arg to weaned pigs enhanced the intestinal immune barrier and maintained intestinal integrity after an *E. coli* lipopolysaccharide (LPS) challenge. These effects were confirmed in suckling piglets that showed an improvement in intestinal barrier function, reduced transepithelial permeability by an increase in villous height in the jejunum by ∼14% and the VH/CD ratio in both the jejunum and ileum by 17% and 25%, respectively (Zheng et al. 2018; Dong et al. 2022). These results may be attributed to the effect of Arg on the oxidative status of piglets, as it enhances NO and nitric oxide synthase (NOS) levels and consequently reduces lactate dehydrogenase (LDH) leakage in the small intestine (Dong et al. 2022). This reduction in LDH leakage could be mediated by the activation of the Arg/NO pathway, as previously observed in the heart (Lin et al. 2008).

In addition, Arg led to a reduction in the inflammation in suckling piglets, notable by a downregulation of proinflammatory cytokine expression of jejunal *IL-1β*, Interferon-gamma (*IFN-γ*), and *IL-2* in comparison to the piglets in the control group before weaning (Dong et al. 2022). Moreover, the piglets in the Arg groups had greater serum levels of IgA, IgG, IgM, and secretory IgA (sIgA, first line of defense of the intestinal epithelium from pathogens) (Tan et al. 2009; Dong et al. 2022) than those in the control group, suggesting an improvement in the piglets’ immune function. Similarly, Zheng et al. (2018) reported a reduction in the inflammatory state of LBW piglets, evidenced by a 32% decrease in IL-1 and a 30% decrease in TNF-α levels in the jejunum. Therefore, Arg supplementation reduced the weaning stress (combined nutritional, social, and environmental challenges associated with separation from the sow, transition to solid feed, and increased inflammation and susceptibility to disease) through the modulation of cytokines.

N-carbamylglutamate (NCG) is a stable analog of N-acetyl glutamate (NAG), which affects pyrroline-5-carboxylate synthase and carbamoyl phosphate synthase-I, both of which influence endogenous intestinal citrulline and Arg synthesis (Li et al. 2023). Zeng et al. (2015) investigated the use of NCG as an alternative to Arg. Piglets supplemented orally with 50 mg/kg BW of NCG during the first 2 wks after birth showed higher ADG compared to those supplemented with Arg. This may be due to increased endogenous synthesis of Arg and the Arg family of AAs in NCG-treated piglets. Similarly, Frank et al. (2007) demonstrated that piglets supplemented with NCG (50 mg/kg BW of NCG every 12 h for 7 d) gained 28% more weight than control pigs. NCG supplementation also influenced intestinal physiology, notably enhancing jejunal mucosal lactase activity (Zeng et al. 2015). Furthermore, NCG significantly increased serum IgA concentrations by 32% and showed a trend to increase sIgA concentration by 15% in the ileal mucosa. Another interesting result obtained with NCG integration was the increase in the total number of cecal *Lactobacillus spp*. and anaerobic bacteria (Zeng et al. 2015), probably since these bacteria can use Arg to grow.

Glutamine (Gln) has been observed to enhance intestinal function and health in weaned piglets (Luise et al. 2022; Luise et al. 2023); however, there is a paucity of data regarding suckling piglets. Only one study was found in present literature research. Schregel et al. (2022) examined the effects of oral Gln supplementation (1 g/kg BW/d) on the growth and function of the jejunum in suckling pigs. In this instance, compared to the control group (isoprotein supplement with the use of alanine), the Gln supplementation in LBW suckling pigs did not result in any change in assessed jejunal parameters. This may imply that oral Gln supplementation is not the best method for promoting jejunum development during the nursing stage. Although glutamine is one of the most abundant AAs in sow colostrum and milk (Gotti et al. 2022), its supplementation has been proposed to support piglet health and robustness due to its functional role (Chalvon-Demersay et al. 2021). Glutamine supports energy metabolism, intestinal integrity, immune function, and oxidative stress reduction, partly through its conversion to glutamate. Moreover, glutamine not utilized by the intestinal environment may be redirected to other metabolic pathways and converted into AAs such as asparagine, alanine, and proline (Ji et al. 2019). In the study of Schregel et al. (2022), piglets were allowed to suckle from the sow while simultaneously receiving Gln supplementation. The results suggest that the glutamine naturally present in sow colostrum and milk was sufficient to meet both the nutritional and functional needs of suckling piglets.

Regarding proteins, a total of five studies investigated their effect on suckling piglets; results are reported in Table 5. Various whey-derived proteins have been incorporated into MRs, with mixed outcomes on physiological development and gut function. Joung et al. (2020) investigated the effects of osteopontin (OPN), a whey protein known to play multiple roles in physiological development, including immune function and gut health (Icer and Gezmen-Karadag 2018). The addition of 250 mg of bovine OPN given via MR to piglets till weaning did not affect BW, ADG, or milk intake. Similarly, OPN supplementation had no significant impact on the concentrations of volatile fatty acids (VFAs) in feces or in the ascending colon (Joung et al. 2020). Only slight numerical changes were observed in the proportions of propionate, valerate, and isobutyrate in the ascending colon, suggesting minor alterations in the gut microbial profile. These findings indicate that bovine milk OPN, when ingested at levels comparable to those naturally present in human milk (100–300 of OPN/L, Schack et al. [2009]), likely remains within the gastrointestinal lumen and undergoes fermentation by colonic microbiota. Results on the β-lactoglobulin (blg) supplementation suggested that even if the blg allowed to increase the DNA quantity in the jejunum mucosa at d 5 post-farrowing, it did not improve the villus height of MR-supplemented pigs compared to sow-reared piglets (Sutton and Alston-Mills 2006). Lactoferrin has been shown to stimulate crypt cell proliferation in neonatal piglets. Reznikov et al. (2014) reported that supplementation with 3.6 g/L of bLF increased crypt cell proliferation by 60% compared to the control group, along with a 20% increase in both crypt depth and area. Wet mucosa weight, length, and villus height in piglets fed bovine colostrum plus porcine plasma (enriched with bovine serum albumin [BSA] and human serum albumin [HSA]) were similar to those fed porcine colostrum in the study by Jensen et al. (2001). However, according to the results of the study by Renshaw and Gilmore (1980) and Jensen et al. (2001), lower intestinal absorption in piglets fed the bovine vs porcine colostrum can be observed; in fact, lower blood circulation of BSA, HSA, protein complement C1–C9 classical (C-CH50) and alternative (except for A-CH50), and lower aminopeptidase activity were observed in the piglets fed the bovine colostrum compared to the ones receiving the porcine colostrum during the first 48 h of life (Renshaw and Gilmore, 1980; Jensen et al. 2001). Interestingly, piglets fed with the bovine colostrum and porcine plasma had a higher activity of maltase in the small intestine. It is not known if bovine colostrum may have a higher concentration of maltase compared to the porcine one; therefore, the results may be due to either the origin of colostrum or the presence of porcine plasma.

**Table 5:** Effects of protein on gut health and growth parameters of suckling piglets.

Additive	Control Group	Group	Dose and timing	Growth performance and health	Epithelial barrier and digestion	Immune fitness	Reference
**Osteopontin**	Milk-deprived piglets fed milk replacer	Milk-deprived piglets + milk replacer + Osteopontin	250 mg/L (ad libitum) till weaning	= BW, ADG, G:F	↓ isobutyrate, propionate ↑ valerate	–	Joung et al. (2020)
**Lactoferrin**	Colostrum-deprived piglets + milk replacer + 0.4 g/L (bLF) × 7–14 d	Colostrum-deprived piglets + milk replacer + Lactoferrin	3.6 g/L × 7–14 d	–	↑ CD, crypt area, crypt proliferation	–	Reznikov et al. (2014)
**β-Lactoglobulin**	Sow-reared piglets	Sow-reared piglets + bovine colostrum + β-Lactoglobulin	10% × 36h + milk replacer till weaning	= weaning BW	↑ DNA quantity (jej)	–	Sutton and Alston-Mills (2006) (exp. 2)
**Bovine serum albumin**	Colostrum-deprived piglets + Porcine colostrum + BSA	Colostrum-deprived piglets + bovine colostrum + BSA	15 mL/kg BW + 50g/L in 12h	–	–	↓ BSA absorption (blood)	Jensen et al. (2001) (exp. 1)
**Porcine plasma + (BSA + HSA)**	Colostrum-deprived piglets + Porcine colostrum + BSA + HSA	Colostrum deprived piglets + bovine colostrum + porcine plasma + (BSA + HSA)	15 mL/kg BW + 50g/L + 20 g/L in 12h	–	= VH ↑ CD, ↑ sucrase, maltase, = lactase, ↓ aminopeptidase IV, = dipeptidyl peptidase, aminopeptidase A	↓porcine IgG, HSA (blood)	Jensen et al. (2001) (exp. 2)

Abbreviations: ADG, average daily gain; BSA, bovine serum albumin; BW, body weight; CD, crypt depth; Ig, immunoglobulin; G:F, gain to feed; has, human serum albumin; Jej, jejunum; VH, villous height

Taken together, these outcomes revealed that the supplementation of AAs or protein sources helps to support piglets’ intestinal morphology and immunological parameters. In particular, Arg is the most investigated AA in suckling piglets and for its role in protein synthesis, NO production, and immune modulation, leading to a reduction in the susceptibility to weaning stress. The protein, mainly derived from bovine milk (OPN, blg, and bLF), seems to have a slight impact on the growth and survival of suckling piglets. Although these results are promising, current evidence remains insufficient to clearly establish their impact on suckling piglets. Further research is needed to determine the optimal dose and timing of administration to maximize the beneficial effects on health.

### Effect of fatty acids, oils, and sugars supplementation

Piglets are born with limited glycogen reserves and high energy demands; therefore, an adequate supply of milk-derived energy is critical for survival, optimal growth, and organ development. Piglets primarily depend on fat and glucose as a source of energy. To investigate the effects of fatty acids (FAs) supplementation in suckling piglets, 5 peer-reviewed articles were analyzed (Table 6), with a focus on long-chain polyunsaturated fatty acids (LCPUFAs) and medium-chain fatty acids (MCFAs). These studies consistently highlight the roles of FAs in energy provision, gut health, immune modulation, and digestive support. Studies on vegetable oils (5, Table 7) and other lipidic sources (4, Table 8) were further analyzed. Fatty acids or oils were administered either diluted in MR (Blank et al. 2002; Wolfe et al. 1977) or directly via oral dosing within the first 24 h of life (Manzke et al. 2018; Schmitt et al. 2019).

**Table 6:** Effects of fatty acid or triglyceride on gut health and growth parameters of suckling piglets.

Category	Control Group	Group	Additive	Dose and timing	Growth performance and health	Epithelial barrier and digestion	Immune fitness	Reference
**Fatty acid**	Milk-deprived piglets + milk replacer	Milk-deprived piglets + Milk replacer + LA-ALA (0.5:1)	Linoleic acid: α-linoleic acid	Ad libitum x 21 days	= weaning BW	–	= MUFA, PUFA n-6, PUFA n-3, EPA	Blank et al. (2002)
		Milk-deprived piglets + Milk replacer + LA-ALA (1:1)					↑ MUFA, PUFA n-6 ↓ PUFA n-3, EPA	
		Milk-deprived piglets + Milk replacer + LA-ALA (2:1)					↑ MUFA, PUFA n-6, DHA ↓ PUFA n-3, EPA	
		Milk-deprived piglets + Milk replacer + LA-ALA (4:1)					↑ MUFA, PUFA n-6, DHA, insulin; ↓ PUFA n-3, EPA	
		Milk-deprived piglets + Milk replacer + LA-ALA (10:1)					↑ MUFA, PUFA n-6; ↓ PUFA n-3, EPA; = DHA	
	Sow-reared piglets (LBW)	Sow-reared LBW piglets + FAs	Caproic acid, Caprylic acid, Capric acid, Lauric acid, Myristic acid, Palmitic acid, Oleic acid, Linoleic acid, Linolenic acid	3g (at birth)	↓ weaning BW; = weaning ADG, mortality; = mortality (d3)	–	–	Declerck et al. (2016)
	Sow-reared piglets (VLBW)	Sow-reared VLBW piglets + FAs		3g (at birth, 12 h)	↓ weaning BW, ADG, mortality (d3); = weaning mortality			
	Milk-deprived piglets + Milk replacer	Milk-deprived piglets + Milk replacer + ARA	ARA	milk replacer + 0.5% × 10 days	= weaning BW	= VH, CD (ile)	ileum: ↑ PUFA, 3H-mannitol, 14C-insuline; = COX-2	Jacobi et al. (2012)
				milk replacer + 5% × 10 d		= VH, CD (ile); = denuded villi surface area (ile)	ileum: ↑ PGE2, PUFA; ↓ 3H-mannitol, 14C-insuline; = COX-2	
		Milk-deprived piglets + Milk replacer + EPA	EPA			= VH, CD (ile)	Ileum: ↓ PGE2, ↑ COX-2, 3H-mannitol, PUFA; = 14C-insuline	
**Triglyceride**	Colostrum-deprived IUGR piglets + milk replacer	Colostrum-deprived IUGR piglets + milk replacer + tributyrin	Tributyrin	0.1% × 14 days	↑ Weaning BW, ADG ↓ diarrhea	↑ VH: CD, ↓ CD, = VH, villous surface area (duo) ↑ lactase, = sucrase, maltase, lipase (duo); = lactase, ↑ trypsin (jej); ↑ lactase, maltase, lipase, trypsin, ↓ sucrase (ile)	↑ IgG, sIgA (ile)	Dong et al. (2016)
**Glycerol**	Sow-reared piglets (LBW)	Sow-reared piglets (LBW) + energy booster	Energy booster: glycerol (898 g/kg), potato protein (99 g/kg), and Vit. E (3 g/kg; EN)	2 mL in 12h	= BW, ADG, ↓ mortality	–	–	Muns et al. (2017)

Abbreviations: ARA, arachidonic acid; ADG, average daily gain; BW, body weight; CD, crypt depth; COX, cyclooxygenase; DHA, docosahexaenoic acid; Duo, duodenum; EPA, eicosapentaenoic acid; Ig, immunoglobulin; Ile, ileum; IGF-1, insulin growth factor-1; IL, interleukin; Jej, jejunum; MUFA, monounsaturated fatty acids; PGE2, prostaglandin; PUFA, polyunsaturated fatty acids; sIg, secretory immunoglobulin; VH, villous height.

**Table 7:** Effects of vegetable oil on gut health and growth parameters of suckling piglets.

Control Group	Group	Dose and timing	Growth performance and health	Immune fitness	Oxidative stress homeostasis	Reference
**Sow-reared piglets**	Milk-deprived piglets + Milk replacer + canola oil + Vit. E	25% canola oil + 16 mg Vit. E/kg milk × 28 d	↑ weaning BW	–	↓ tocopherol α, ↑ tocopherol γ+δ (heart)	Sauer et al. (1997) (Exp.1)
	Milk-deprived piglets + Milk replacer + soybean oil + Vit. E	25% soybean oil + 16 mg Vit. E/kg milk × 28 d			↓ tocopherol α, ↑ tocopherol γ+δ	
	Milk-deprived piglets + Milk replacer + olive oil + Vit. E	25% olive oil + 16 mg Vit. E/kg milk × 28 d			↑ tocopherol α, γ+δ	
	Milk-deprived piglets + Milk replacer + canola: coconut + Vit. E	25% canola:coconut oil + 16 mg Vit. E/kg milk × 28 d			↓ tocopherol α, ↑ tocopherol γ+δ	
	Milk-deprived piglets + Milk replacer + canola oil + Vit. E	25% canola oil + 93 mg Vit. E/kg milk × 28 d	= weaning BW	–	↑ tocopherol α, γ+δ	Sauer et al. (1997) (Exp.3)
	Milk-deprived piglets + Milk replacer + soybean oil + Vit. E	25% soybean oil + 93 mg Vit. E/kg milk × 28 days				
	Milk-deprived piglets + Milk replacer + high oleic acid sunflower oil + Vit. E	25% high oleic acid sunflower oil + 93 mg Vit. E/kg milk × 28 d			↑ tocopherol α, ↓ tocopherol γ+δ	
	Milk-deprived piglets + Milk replacer + high oleic acid sunflower oil + soybean oil + linseed oil + Vit. E	25% high oleic acid sunflower oil + soybean oil + linseed oil + 93 mg Vit. E/kg milk × 28 d			↑ tocopherol α, γ+δ	
	Sow-reared piglets + Rice bran oil	2 mL × 2 (at birth and 24 h)	= weaning BW, ADG	–	–	Manzke et al. (2018) (Exp.1)
		4 mL × 2 (at birth and 24 h)				
		8 mL × 2 (at birth and 24h)				
		16 mL × 2 (at birth and 24 h)				
	Sow-reared piglets + Rice bran oil + canola oil, sunflower oil, and corn oil	2 mL × 2 (at birth and 24 h)	= weaning BW, ADG	= triglycerides (8 h, d 7)	–	Manzke et al. (2018) (Exp.3)
	Sow-reared piglets + Glycerin	2.33 mL × 2 (at birth and 24 h)		↑ triglycerides (8 h) = triglycerides (d 7)		
	Sow-reared piglets + Soybean oil	1.3 mL × 2 (at birth and 24 h)		= triglycerides (8 h, d 7)		
	Sow-reared piglets + Linseed oil	1.4 mL × 2 (at birth and 24 h)		= triglycerides (8 h, d 7)		
	Sow-reared piglets + Coconut oil	1.68 mL x 2 (at birth and 24 h)		= triglycerides (8 h) ↓ triglycerides (d 7)		
	Sow-reared piglets + Rice bran oil	2 mL × 2 (at birth and 24 h)		= triglycerides (8 h) ↑ triglycerides (d 7)		
**Sow-reared piglets (LBW) + water**	Sow-reared piglets (LBW) + product enriched in MCFA	2 mL (after 3 h)	= weaning BW, ADG, mortality	= Glucose	–	Schmitt et al. (2019)
	Sow-reared piglets (LBW) + coconut oil		↑ weaning ADG; = weaning BW, mortality	↑ Glucose		

Abbreviations: ADG, average daily gain; BW, body weight.

**Table 8:** Effect of energy sources from animal sources on gut health and growth parameters of suckling piglets.

Additive	Control group	Group	Dose and timing	Growth performance and health	Immune fitness	Oxidative stress homeostasis	Reference
**Fish oil**	Sow-reared piglets	Sow-reared piglets + Infant formula + fish oil	1.5% of total lipids of the infant formula × 14 d	–	–	↓ DHA, ↑ AA (Blood, small intestine), ↑ EPA (Small intestine)	Goustard-Langelier et al. (1999)
			4.5% of total lipids of the infant formula × 14 d			↓ DHA, EPA, ↑ AA (Blood, small intestine)	
**Egg yolk phospholipids**		Sow-reared piglets + Infant formula + egg yolk phospholipid	17% of total lipids of the infant formula × 14 d				
**Fish oil**		Sow-reared piglets + Infant formula + salmon oil	3% of total FA × 14 d	–	–	↑ AA, EPA, DHA	Alessandri et al. (1998)
		Sow-reared piglets + Infant formula + low-EPA fish oil	1.5% of total FA × 14 d			↑ AA, DHA; = EPA	
			4.5% of total FA × 14 d			↑ EPA, DHA; ↓ AA	
**Egg yolk phospholipids**		Sow-reared piglets + Infant formula + egg yolk phospholipids	17% of total FA × 14 d			↑ DHA; ↓ EPA, AA	
**Butter fat**	Colostrum-deprived piglets + butterfat 2% liquid diet	Colostrum-deprived piglets + butter fat 17% liquid diet	17% BF ad libitum × 14 d	↑ ADG (d14)	–	= fatty acid synthetase, citrate cleavage enzyme, malic enzyme (Liver, adipose tissue)	Wolfe et al. (1977)
		Colostrum-deprived piglets + butter fat 32% liquid diet	32% BF ad libitum × 14 d				
**Fat**	Colostrum-deprived piglets + 30 mL of Ig-free milk replacer	Colostrum-deprived piglets + colostrum supplement (fat, carbohydrate, porcine IgG)	30 mL/4 h × 15 d	↑ Weaning BW, ADG ↓ mortality	↑ IgG (blood)	–	Polo et al. (2012)

Abbreviations: AA, amino acid; ADG, average daily gain; BW, body weight; DHA, docosahexaenoic acid; EPA, eicosapentaenoic acid; Ig, immunoglobulin.

When piglets were fed ad libitum with an MR enriched with different ratios of linoleic acid (LA) and alpha-linolenic acid (ALA), where LA remained constant and ALA varied (0.5:1–10:1), no differences in BW at weaning were observed (Blank et al. 2002). The same result was also found when MR was enriched with arachidonic acid (ARA) and eicosapentaenoic acid (EPA), respectively, at 0.5% and 5% at d 10 of life (Jacobi et al. 2012). This can be explained by the fact that both studies involved isoenergetic diets, meaning the energy content of the diets was equal. Similarly, in IUGR piglets, Dong et al. (2016) showed that MR enriched with 0.1% tributyrin from d 7 to d 21 of life improved BW and ADG on d 21, respectively, by 34.45% and by 191% compared to the IUGR piglet group that did not receive the supplement; in fact, tributyrin improved the growth, the digestive, and the immune functions of piglets (Dong et al. 2016). In cases of LBW and VLBW piglets, giving them a dose of 3 g of a commercial product composed of MCFAs immediately after birth, or a double dose (3 g after birth and 3 g after 8–12 h), the weaning weight (d21) was lower than the control group, while ADG values were not different (Declerck et al. 2016). The lower weaning BW was attributed to the low birth BW of the piglets (Gondret et al. 2005). Similar results were reported by Muns et al. (2017) when supplementing an energy booster enriched in glycerol (glycerol [898 g/kg], potato protein [99 g/kg], and Vit. E [3 g/kg; EN]), which helped in maintaining growth parameters and reduced mortality rate in LBW piglets.

The analysis of the FAs in the plasma of piglets showed that, with an increase in LA:ALA ratio in the diet, the levels of EPA decrease, while levels of ARA increase (Blank et al. 2002). This process is due to the competition between the precursors of EPA and ARA, n-3 and n-6 PUFAs, for the Δ6-desaturase and Δ5-desaturase enzymes, which are essential for desaturation reactions (Voss et al. 1991). A linear increase in docosahexaenoic acid (DHA) levels in the plasma was also observed up to an LA:ALA ratio of 1:4 (Blank et al. 2002), beyond which DHA levels decreased due to the inhibition of the conversion of n-3 PUFAs (particularly tetracosapentaenoic acid) into DHA caused by high ALA concentrations (Geiger et al. 1993).

Regarding intestinal morphology, the addition of ARA (0.5%) or EPA (5%) in the MR appeared not to affect the intestinal structure at the ileum level of weaned piglets (Jacobi et al. 2012), but when piglets were subjected to an ischemic challenge and fed with 5% ARA, there was a reduction by 30.16% of the denuded villus surface in the ileum compared to the control group. This indicates greater protection of the denuded villous surface under ischemic stress conditions in piglets. Additionally, an improvement in nutrient absorption and digestion was identified, with a significant increase in the enzymatic activity of lactase, maltase, and trypsin in IUGR piglets fed with tributyrin (0.1%) (Dong et al. 2016). Furthermore, tributyrin had an enhanced effect of IgG and sIgA at the ileum level.

Piglets fed with MR containing 25% of LCPUFAs from various vegetable sources (canola oil, soybean oil, olive oil, and mix of canola and coconut oils) and enriched with 16 mg/kg of vitamin E, showed an increasing BW (36.1%) and ADG (51.65%) at weaning (d28) compared to the control group that remained with the sow (Sauer et al. 1997). However, when FAs are not administered with ad libitum feed but are provided directly to the piglets in a measured dose, occasionally, no benefits in the growth performance were observed. In fact, when the piglets were fed with 2 mL of coconut oil 3 h after birth, compared to the control group, no effects were observed (Schmitt et al. 2019). Manzke et al. (2018) observed that administering a single dose of rice bran oil (rich in LCFA) to newborn piglets at different doses (from 2 mL to 16 mL), it does not change the glucose and triglyceride levels at 8 h after the ingestion. However, with a double dose of 2 mL (at birth and 24 h), triglyceride levels increased by 44.24% at 8 h and 27.22% on d 7 compared to the control group. Thus, rice bran oil serves as a lasting energy source for piglets during lactation (Manzke et al. 2018). In the case of administering 2 mL of coconut oil at 3 h of life (Schmitt et al. 2019), no difference was recorded in the glucose levels of plasma immediately after administration or 24 h later.

Comparing the collected data on intestinal morphology, it was observed that in the small intestine, both villus height and crypt depth show no differences due to the administration of different energy sources, such as rice bran oil, soybean oil, or linseed oil diluted in MR administered to piglets (Manzke et al. 2018). This suggests that the different sources of LCPUFA did not change the intestinal structure in the first weeks of piglet life.

Fat was also tested as a potential strategy in the study conducted by Wolfe et al. (1977). Authors showed an increase in the ADG (d 14 of life) in colostrum-deprived piglets fed exclusively with MR enriched with butterfat, at 17% or 32%, by 16% and 25%, respectively, compared to the group that received MR without any butterfat content. Although the diets were isoenergetic (glucose was gradually replaced with butterfat), the findings indicate that energy derived from butterfat is utilized with comparable efficiency to that from glucose in supporting growth. Furthermore, an increased weaning weight and ADG and a decrease in mortality rate were observed in colostrum-deprived piglets when fed a colostrum supplement (30 mL/4h x 15 d) enriched with energy sources (fat and carbohydrate) (Polo et al. 2012).

The study by Polo et al. (2012) was the only one to analyze Igs variation and observed an increase of IgG concentration at 24 h post-administration of 30 mL of colostrum supplement enriched with fat, carbohydrates, and IgG from porcine plasma in piglets (7.6 vs 0.14 mg/mL), namely due to the integration of Igs in the colostrum supplement.

The levels of EPA in plasma depend on the dietary amount and the origin of the raw material. In piglets fed with infant formula enriched with salmon oil (0.3% of EPA) and a diet enriched with 4.5% fish oil (low-EPA), the blood EPA levels were significantly higher than in sow-reared piglets (Alessandri et al. 1998). The study by Alessandri et al. (1998) demonstrated a significant increase in DHA levels in plasma phospholipids, particularly in groups that received infant formula with 4.5% fish oil and egg yolk phospholipids, showing a DHA increase of 98.31% and 59.32%, respectively (Alessandri et al. 1998; Goustard-Langelier et al. 1999).

High concentrations of DHA in piglets’ intestines fed with different FA sources (salmon oil, fish oil, egg yolk phospholipids) (Goustard-Langelier et al. 1999), and a significant increase in EPA concentrations (133%) were recorded in piglets fed a formula enriched with 4.5% fish oil. Effective absorption of DHA in the intestine is observed with fish oil supplementation. However, the ARA concentration in the group supplemented with fish oil was 10% lower than in the control group, suggesting that fish oil may reduce ARA at the intestinal level, especially when DHA is provided at high concentrations in the diet. The variations in DHA, EPA, and ARA observed indicate that the different incorporation of FA based on various dietary sources highlights the importance of balancing the n-3 and n-6 LCPUFA ratio for an optimal available fatty acid composition in the intestine for the piglets (Goustard-Langelier et al. 1999). In contrast, supplementation with egg yolk phospholipids maintained a balance between EPA and ARA, like that observed in the control group, indicating a balanced effect between these FAs.

Besides the use of FAs and fat-based ingredients, another strategy to supply additional energy to neonatal piglets is the administration of simple sugars. These have been mostly tested initially via injectable routes with positive effects. In fact, Engelsmann et al., 2019 reported that, in IUGR sow-reared piglets supplemented with 6 mL of glucose via four 1.5 mL injections (two in the groin and two in the neck) at 0, 3, and 6 h of life, an increase in weaning BW (d 21) by 12% and 10%, respectively, and in ADG by 13% and 12%, respectively, compared to untreated IUGR piglets, can be obtained. Moreover, supplementation with glucose reduced mortality by 50%, suggesting that an early extra dose of energy can improve robustness and competitiveness in these compromised piglets.

The search of the present review allowed us to find two articles in which sugar was orally supplemented to suckling piglets: one about glucose and one about dextrose equivalent (DE) supplementation.

Analyzing the piglets’ growth performance, Jarratt et al. (2023), who tested the glucose at birth (300 mg), observed no significant differences in BW between groups until weaning (5.88 kg vs 5.83 kg; 248 g/d vs 247 g/d), except for d 3 of life, when the control group weighed more (7%) than the glucose one. When piglets were analyzed by birth BW, those with LBW that received both glucose and caffeine (300 mg plus 30 mg, respectively) supplementation exhibited growth rates comparable to the LBW control group (at d 3 of life) and higher than those of piglets supplemented with either caffeine or glucose alone, likely due to the combined effects of glucose as an immediate energy source and caffeine as a metabolic stimulant (Jarratt et al. 2023). Similarly, replacing lactose with dextrose or corn syrup solids in piglets’ diets did not improve weaning BW (d 20), although dextrose proved to be an effective lactose substitute (Oliver et al. 2002). Oral administration appears to have a more limited effect than sugar injection; in fact, glucose injections provide easily and rapidly usable energy, bypassing intestinal absorption, thereby preventing hypoglycemia and maintaining blood levels above 2.8 mmol/L, as reported by Goodwin (1957).

Early administration of energy sources (fat and sugar), particularly within the first 24–48 h after birth, is considered critical for improving vitality, supporting growth, and reducing neonatal mortality. However, most studies have focused primarily on the metabolic utilization of these energy sources, while parameters related to intestinal health have received limited attention. In any case, as with other additives, the timing of administration and the different energy sources, including fats and sugars, have yielded promising results on growth performance and gut health.

### Effect of mineral and vitamin supplementation

When analyzing the effects of mineral supplementation in suckling piglets, the retained articles provided limited investigation of gut health parameters and were mainly focused on growth performance (Table 9). Two articles on calcium supplementation in piglets’ formula were found (Wauben and Atkinson 1999; Wan et al. 2016). Calcium supplementation is a common practice for human premature infants fed their mother’s milk, as colostrum and milk are deficient in this mineral, which is important to prevent osteopenia (Schanler 1995).

**Table 9:** Effects of vitamins and minerals on gut health and growth parameters of suckling piglets.

Category	**Control group**	**Group**	**Additive**	**Dose and timing**	**Growth performance and health**	**Immune fitness**	**Oxidative stress homeostasis**	**Reference**
**Mineral **	Milk-deprived piglets + milk replacer	Milk-deprived piglets + Milk replacer + Calcium	Calcium	4.67 g/L Ca × 21 d	= BW	= iron absorption	−	Wauben and Atkinson (1999)
		Milk-deprived piglets + Milk replacer + β-hydroxy-β-methyl butyrate calcium	Β-hydroxy-β-methyl butyrate calcium	800 mg/kg × 21 d	= weaning BW, ADG, FCR	↑ insulin, leucine, BCAA, EAA, NEAA (plasma) = urea (plasma) ↑lactate dehydrogenase	−	Wan et al. (2016)
**Vitamin **	Sow-reared piglets + Bovine milk	Whole milk + Vit. C	Vitamin C	75 ppm	= BW, ADG	↓cortisol, TNF-α (plasma) ↑IL-1β, TNF-α, IL-1Ra (intestine)	–	Eicher et al. (2006) (LPS challenge)
		Whole milk + Vit. C + β-glucans	Vitamin C + β-glucans	75 ppm + 0.312 g/kg BW	↑ BW, ADG	= cortisol (plasma)		
**Mineral + Vitamin **	Bovine colostrum	Bovine colostrum + Vit. A + Vit. D + Cu	Vit. A + Vit. D + copper	200-400 g/litter + Vit A [23,250 IU (d2) and 46,500 IU (d5)] + Vit. D [4000 IU (d2) and 8,000 IU (d5)] + copper [4mg (d2) and 8 mg (d5)] till weaning	= weaning BW, ADG	−	= SOD, GPX, MDA, FRAP, CAT	Galiot et al. (2021)

Abbreviations: ADG, average daily gain; BCAA, branched chain amino acid; BW, body weight; CAT, catalase; EAA, essential amino acid; FRAP, ferric reducing antioxidant power; GPX, glutathione peroxidase; IL, interleukin; MDA, malondialdehyde; NEAA, non-essential amino acids; SOD, superoxide dismutase; TNF, tumor necrosis factor.

Regarding growth, administering 4.67 g/L of calcium glycerophosphate (CaGP) from d 7 to d 21 after farrowing (Wauben and Atkinson 1999) or 800 mg/kg of HMB-Ca (β-hydroxy-β-methyl butyrate calcium) administered from d 7 to d 28 of life (Wan et al. 2016) did not improve the BW and ADG of suckling piglets. However, considering IUGR piglets, the administration of 800 mg/kg of HMB-Ca allowed to achieve the same BW, ADG, and FCR as normal piglets without the Ca supplementation (Wan et al. 2016). The piglets supplemented with HMB-Ca also exhibited a greater abundance of skeletal muscle and maturation of the muscle fiber growth. Skeletal muscle is 30% of the body mass in suckling piglets (Davis and Fiorotto 2009); therefore, this result is strictly related to the increase in BW observed in the study. Furthermore, in the same study, the HMB-Ca led to an increased plasma circulation of insulin, leucine, branched-chain amino acids (BCAA), and non-essential amino acids (NEAA), which are related to transcription (ie, mTOR pathway) and growth factors that stimulate muscle and fiber maturation (Wan et al. 2016).

Regarding vitamin supplementation in suckling piglets, two articles were retained: one regarding the administration of vitamins A and D (Galiot et al. 2021) and one of vitamin C (Eicher et al. 2006).

The administration of vitamins A and D in the form of retinol acetate and 25-OH-D3 (calcifediol) through two interventions, one on d 2 with doses of 23,250 IU and 4,000 IU, respectively, and one on d 5 with doses of 46,500 IU and 8,000 IU, respectively, did not improve piglets’ performance. However, if an extra dose of bovine colostrum is added, a slight increase in both BW and ADG at weaning was observed, respectively by 2.67% and 2.57% (Galiot et al. 2021). Comparisons of the same groups of piglets that were born from sows that received certain micronutrients, such as copper and retinol acetate, showed better performance at weaning, higher glutathione peroxidase (GP×2), and lower activities and malondialdehyde (MDA) in the blood. This result suggests that paired feeding strategies for piglets and sows can be successful. Maintenance of growth performance has been reported by Eicher et al. (2006), in which the administration of 75 ppm of vitamin C alone or combined with 0.312 g of β-glucan diluted in 10 mL of whole milk and administered in a single dose from d 1 to d 13 of life, resulted in the same (vitamin C) or higher (vitamin C + β-glucan) BW and ADG at weaning compared to the control group.

Regarding the immune status pillar, Galiot et al. (2021) reported a 30% reduction in plasma ferric reducing antioxidant power (FRAP) in piglets supplemented with a combination of vitamins and copper. Lower plasma concentrations of TNF-α were detected in early-weaned piglets (14 d old) that received 75 ppm of vitamin C diluted in 10 mL of whole milk throughout the lactation period and were challenged with LPS (Eicher et al. 2006). This reduction may be attributed to decreased cortisol levels and a diminished cytokine release, indicating a moderate response of the hypothalamic–pituitary–adrenal (HPA) axis. In the same study, intestinal concentrations of IL-1β, TNF-α, and IL-1 receptor antagonist (IL-1Ra) increased by 209%, 455%, and 159%, respectively, in piglets supplemented with vitamin C alone. In contrast, in organs such as the liver, lungs, and spleen, higher TNF-α levels were detected across all treatment groups compared to the control. Notably, piglets supplemented with vitamin C alone or in combination with β-glucans exhibited an increase in IL-1Ra expression in the liver.

Vitamin and mineral supplementation through MRs helped maintain the oxidative balance of piglets and had a positive effect on growth performance.

### Effect of probiotics and prebiotics

#### Probiotic

The neonatal period is an effective window of intervention for the modulation of the intestinal microbiota via the use of pro- and prebiotics, thanks to the rapid and dynamic settlement of the bacteria (Trevisi et al. 2021). It is important to highlight that sow colostrum and milk have their own microbiota and that the presence of environmentally derived bacteria is almost inevitable under farm conditions (Luise et al. 2025). In particular, Martín et al. (2009) reported a high prevalence of potentially beneficial bacteria, such as lactic acid bacteria in milk samples, including *Lactobacillus reuteri, L. salivarius, and L. paraplantarum*, which are known for their strong probiotic effects.

Data on probiotic and prebiotic supplementation are reported in Tables 10 and 11, respectively.

**Table 10:** Effects of probiotics on gut health and growth parameters of suckling piglets.

Probiotic	Control group	Group	Dose and timing	Growth performance and health	Epithelial barrier and digestion	Immune fitness	Microbiota	Reference
***Lactobacillus sp.***	Sow-reared piglets + Milk replacer	Sow-reared piglets + milk replacer + Probiotic	1 × 10^9^ CFU/g × 21 d	↑ BW, ADG, = ADFI	–	–	↑ *Lactobacillus, Bacteroidota*	Sampath et al. (2023)
***Lactobacillus fermentum***		Milk-deprived piglets + milk replacer + probiotic	6 × 10^9^ CFU/g × 14 d	= BW ↑ ADG	↑ VH, butyrate, isobutyrate, isovalerate	↓ *IL-1β*	↓ Clostridium sp.	Liu et al. (2014)
***Lactobacillus paracasei***	Colostrum-deprived piglets + milk replacer	Colostrum-deprived piglets + milk replacer + probiotic	2.6 × 10^8^ CFU/kg × 5 d	= ADG	= Acetate, butyrate, SCFAs	–	–	Andersen et al. (2017)
***Pediococcus pentosaceus***			1.3 × 10^10^ CFU/kg × 5 d	= ADG	↑ Acetate, butyrate, = SCFAs			
***Pediococcus acidilactici***	Sow-reared piglets + sterile saline	Sow-reared piglets + Sterile saline + probiotic	1 × 10^9^ CFU/g × 7 d	↑ BW, ADG ↓mortality	–	↑ TGF-β1, IgA, = IgG	–	Sarkar et al. (2023)
***Saccharomyces cerevisiae boulardi***	Sow-reared piglets + water	Sow-reared piglets + Water + probiotic	1 x 10^10^ CFU/g × 7 d	↑ BW, ADG ↓mortality	–	–	*↑ Erysipelatoclostridium, Christensenella*	(Luise et al. 2021b)
***Enterococcus faecium lactiferm***			1 × 10^10^ CFU/g × 7 d	↑ BW, ADG = mortality			*↑ Lachnospiraceae*	
***Saccharomyces cerevisiae boulardi + Enterococcus faecium lactiferm***			1 × 10^10^ CFU + 1 x× 10^10^ CFU × 7 d	= BW, ADG, mortality				
***Saccharomyces cerevisiae***			5 x 10^9^ CFU/pig (3 mL) × 28 d	↑ BW, ADG	–	–	*↑ Blautia, Collinsella, Eubacterium*	Kiros et al. (2019)
			2.5 x 10^10^ CFU/pig (6 mL) × 28 d				*↑ Eubacterium, Anaerostipes, Parabacteroides, Mogibacterium, Phascolarctobacterium*	
***Enterococcus faecium* + *Saccharomyces cerevisiae*, inulin, immunoglobulins, vitamins, selenium**		Sow-reared piglets + Water + symbiotic	2 mL at birth (10 h)	↑ ADG (d0-d39) ↑ BW ↓ Post-weaning diarrhea	= SCFAs	–	↑ *Lactobacillus*	Girard et al. (2021)

Abbreviations: ADG = Average Daily Gain, BW = Body Weight, Ig = Immunoglobulin, IL = Interleukin, SCFAs = Short-Chain Fatty Acids, TGF-β = Transforming Growth Factor, VH = Villous Height.

**Table 11:** Effects of prebiotics on gut health and growth parameters of suckling piglets.

Prebiotic	Control group	Group	Dose and timing	Growth performance and health	Epithelial barrier and digestion	Immune fitness	Microbiota	Reference
**Fructo-oligosaccharides**	Sow-reared piglets + Water	Sow-reared piglets + Water + prebiotic	1g/day (d0–d7)	↑ BW ↓post-weaning mortality	= VH, CD = SCFA	↑ IFN-γ = IgG	–	Ayuso et al. (2020)
			1g/day (d0–d21)	–	–	= IFN-γ, IgG		
**Galacto-oligosaccharides + polydextrose**	Sow-reared piglets + Milk replacer	Sow-reared piglets + Milk replacer + oligosaccharides	1 g/day × 7 d	= BW ↑ mortality	–	–	–	Van Tichelen et al. (2021)
	2 days-old piglets + Milk replacer	2 days-old piglets + Milk replacer + oligosaccharides	2 g/L × 19 d	= BW	= VH, CD ↓ propionate, = isovalerate (ile), isobutyrate, isovalerate (colon)	–	↑ *Lactobacillus*	Hoeflinger et al. (2015)
**2'-fucosyllactose + lacto-N-neotetraose + 6'-sialyllactose + 3'-sialyllactose + free sialic acid**	Milk-deprived piglets + milk replacer	Milk-deprived piglets + Milk replacer + lactose	4g/L × 14 d	↓ Diarrhea	–	↓ IL-8, ↑ IFN-γ, IL-10	↑ *Lachnospiraceae*	Li et al. (2014) (rotavirus challenge)
**Short-chain galacto-oligosaccharides + long-chain fructo-oligosaccharides/L**		Milk-deprived piglets + Milk replacer + prebiotic	3.6 g + 0.4 g/L × 14 d			= IL-8, IL-10, IFN-γ	–	
**2'-fucosyllactose, lacto-N-neotetraose, 6'-sialyllactose, 3'-sialyllactose + free sialic acid**	Colostrum-deprived piglets + Milk replacer	Colostrum-deprived piglets + Milk replacer + Lactose + prebiotic	4g/L × 15 d	= BW, ADG	–	↑ IFN-γ ↓ PBMC	–	Comstock et al. (2017)
**Short-chain galacto-oligosaccharides + long-chain fructo-oligosaccharides**			3.6 g + 0.4 g/L × 15 d			= IFN-γ, PBMC		
**Polydextrose + galacto-oligosaccharides + bovine lactoferrin + milk fat globule membrane**	Colostrum-deprived pigs + milk replacer	Colostrum-deprived pigs + milk replacer + prebiotic	1.2 g + 3.5 g+ 0.3 g + 2.5 g/100 g × 30 d	↑ BW	↑ Lactase (jej), tyrosine hydroxylase (duo), vasoactive intestinal peptide (ile)	–	↑ *Bacteroidetes* ↓ *Proteobacteria*	Berding et al. (2016)

Abbreviations: ADG, average daily gain; AST, aspartate aminotransferase; BW, body weight; CD, crypt depth; Ig, immunoglobulin; IL, interleukin; Ile, ileum; IFN, interferon; PBMC, peripheral blood mononuclear cell; SCFAs, short-chain fatty acids; TGF-β, transforming growth factor; VH, villous height.

The search allowed to retain six studies investigating the effect of probiotic supplementation on suckling piglets. The studies mainly considered probiotics belonging to the Lactobacillaceae family (four studies), and to the families of Saccharomycetaceae and Enterococcaceae (two studies).

Regarding the Lactobacillaceae family, the genera *Lactobacillus a*nd *Pediococcus* were mainly investigated. These probiotics can be used either alone (Sampath et al. 2022), in combination with other probiotics from different families (Siggers et al. 2008), or together with prebiotics, known as symbiotics (Girard et al. 2021). Administration occurred orally, and the probiotics were provided directly to the piglet by gastric tube (Andersen et al. 2017) or diluted in MR (Sampath et al. 2022). An analysis of growth performance indicated that piglets supplemented with *Lactobacillus* spp. at a dose of 1 × 10^9^ CFU/g/day, administered every 3 h and diluted in MR from d 3 to d 21 of life, exhibit significantly higher BW and ADG compared to non-supplemented piglets (Sampath et al. 2022). A similar improvement in growth performance could also be achieved through the administration of multi-strain probiotic formulation. In the context of delayed weaning, Sarkar et al. (2023) reported that the administration of *Pediococcus acidilactici* FT28 (2 mL, 1 × 10^9^ CFU/g) during the first week of life resulted in significantly higher BW and ADG at weaning (d 42) compared to suckling piglets from dams. Moreover, the study observed a significant increase in piglet survival during the lactation period. These findings were likely due to an enhanced immune response, as indicated by higher serum concentrations of IgG, IgA, and TGF-β1. This immune response is associated with improved epithelial barrier integrity, ultimately contributing to better growth performance. Liu et al. (2014) demonstrated that oral administration of *Lactobacillus fermentum* I5007 (3 mL), diluted in peptone water from d 1 to d 14, reported an improvement of the villus height, enhanced nutrient absorption, and increased butyric acid production, which is associated with improved intestinal function, with a reduced incidence of diarrhea. Additionally, piglets supplemented with *L. fermentum* I5007 exhibited significantly lower levels of IL-1β and IL-10, by 53.85% and 30.77%, respectively, indicating reduced intestinal inflammation. Controversial results were obtained by Andersen et al. (2017) by supplementing *Lactobacillus paracasei* to newborn cesarean-delivered colostrum-deprived term piglets; in fact, the probiotic supplementation increased diarrhea in piglets challenged with ETEC F18. This contrasting result may be due to the fact that the piglets were not in normal physiological condition, and the deprivation of colostrum and maternal milk could have diminished the positive effect of the probiotic.

Notably, the supplementation of probiotics also allowed for a reduction in the abundance of potentially pathogenic bacteria such as *Escherichia-Shigella* and Clostridium (Sampath et al. 2022) and coliforms (Shim et al. 2006; Liu et al. 2014). This reduction is linked to a lower risk of digestive disorders and intestinal inflammation, as beneficial bacteria compete for available substrates. Moreover, high concentrations of *Lactobacillus* in the intestine have been correlated with a reduced incidence of diarrhea and improved growth performance in piglets receiving probiotic treatment.

*Saccharomyces cerevisiae* is one of the main strains used as an additive in pigs’ diet; a total of 2 articles testing this strain have been found. Luise et al. (2021b) investigated the potential use of *Saccharomyces cerevisiae* and *Enterococcus faecium* in suckling piglet nutrition. In this study, a single dose of 4 mL (1 × 10^10^ CFU) was administered at two days of life. At weaning (d 21), piglets treated with either probiotic strain exhibited significantly higher BW and ADG compared to the control group (suckling piglets without probiotic supplementation), with increases of 7.6% and 9% in BW and 10.3% and 6.86% in ADG, respectively. Positive outcomes were also obtained by Kiros et al. (2019) by supplementing two different doses of *Saccharomyces cerevisiae* (5 × 10^9^ CFU/pig or 2.5 × 10^10^ CFU/pig) through lactation; both concentrations had an improvement in piglets’ BW and ADG at weaning. The combined administration of *Saccharomyces cerevisiae* and *Enterococcus faecium* in suckling piglets did not result in significant differences in BW or ADG compared to non-supplemented piglets (Luise et al. 2021b). This lack of effect is likely due to competition between *S. cerevisiae* and *E. faecium* for nutrient resources and adhesion sites in the intestinal environment (Chapman et al. 2011). Additionally, probiotic supplementation modulated the intestinal microbiota by enhancing the abundance of beneficial bacterial species. Specifically, *Erysipelatoclostridium* and *Christensenella* were enriched in the *Saccharomyces cerevisiae* group, whereas *Lachnospiraceae* UCG-004, *Sanguibacteroides*, and *Alistipes* were more prevalent in the *Enterococcus faecium* group (Luise et al. 2021b). This microbial modulation contributed to the establishment of a more stable intestinal environment, improving nutrient absorption and feed conversion efficiency. In fact, Kiros et al. (2019) demonstrated that *S. cerevisiae* shaped, in different ways, the cecum microbiota composition, positively influencing their growth. In particular, the group supplemented at a dose of 5 × 10^9^ CFU/pig was mainly characterized by *Blautia, Collinsella*, and *Eubacterium*, while the highest dose of yeast (2.5 × 10^10^ CFU/pig) had a microbial community highly characterized by Eubacterium, Anaerostipes, Parabacteroides, Mogibacterium, and Phascolarctobacterium.

#### Prebiotics

Prebiotics are nondigestible dietary fibers that serve as substrates for the selective stimulation of beneficial gut microbiota, thereby enhancing their growth and metabolic activity. Swine colostrum and milk naturally contain prebiotic compounds, primarily oligosaccharides, although their concentrations vary throughout lactation and breed (Tao et al. 2010; Trevisi et al. 2020). The results of the literature research focused on oligosaccharides with three studies on the *Fructo-oligosaccharides*, five on *Galacto-oligosaccharides*, and two studies on oligosaccharides derived from human milk (Table 11).

#### Fructo-oligosaccharides

Short-chain fructo-oligosaccharides (scFOS) have been classified as prebiotics since they are not digested in the intestinal tract, resulting in a fermentable substrate for the gut microbiota Sridevi et al. 2014). They have been shown to benefit piglets’ health by inducing a shift in the gut microbiota, favoring *Bifidobacteria* and *Lactobacillus* spp. (Schokker et al. 2018), which efficiently digest them into SCFA and are favored over potentially harmful bacteria like *Clostridium perfringens*, *Clostridium difficile*, *Escherichia coli*, and *Streptococcus thermophilus*, limiting their adhesion to the mucosa.

Ayuso et al. (2020) observed that ScFOS supplementation (1 g/d) did not impact growth performance or pre-weaning mortality at the end of the experiment. Despite the absence of effects while breastfeeding, one of the most noteworthy findings was a considerable reduction in post-weaning mortality after 7 or 21 d of scFOS supplementation, which reached 0%. Moreover, scFOS boosted the neonatal immune system by raising IgA production and activating T-cells, and IFN-γ levels.

Conflicting results were obtained by Van Tichelen et al. (2021), who showed that not all the beneficial effects of scFOS were confirmed when given to LBW piglets. In fact, there was no influence on BW or biochemical blood values, such as glucose, NEFA, urea, IgG, and IGF-1 among groups. In this case, scFOS did not improve LBW piglets’ performance or health and had a detrimental impact on survival rates; in fact, the cumulative death rate was greater in piglets in the scFOS group than in the MR group. These negative outcomes could partly be attributed to the supplementation period (7 d), which could have been too short or too long, as well as to the extremely LBW (860 g) of the piglets, suggesting that BW may play an important role in the efficacy of scFOS. In fact, LBW piglets suffer from severe intestinal immaturity (Trevisi et al. 2023), which may also have contributed. An underdeveloped gut barrier and unstable microbiota could lead to excessive or unbalanced fermentation of scFOS, increased osmotic load, impaired nutrient absorption, and a higher risk of dysbiosis, potentially compromising survival. However, since these physiological and microbial aspects were not specifically investigated in that study, the underlying mechanisms remain largely unclear.

#### Galacto-oligosaccharides

Galacto-oligosaccharides (GOS) are galactose-containing oligosaccharides whose chemical structures vary by chain length, branching, and glycosyl linkages. Hoeflinger et al. (2015) focused their study on the modification in the gut microbial population of piglets fed bovine MR formula supplemented with GOS and polydextrose (2 g/L × 19 d). Among the study’s findings, the piglets’ final BW and overall ADG were not significantly different between groups, while Berding et al. (2016) observed an increase in BW when GOS was implemented with bLF and milk fat globule membrane.

Furthermore, intestinal histomorphology revealed no significant differences between the groups in ileal villus length, crypt depth, or total presumptive *Lactobacilli* count (CFU/g). In this context, the intestinal bacterial population changed between treatments, showing that Parabacteroides and *Lactobacillus* spp. were significantly more abundant in the group supplemented with polydextrose. So, the study found that using GOS directly did not lead to an increase in *Lactobacillus* spp. Different kinds of results were obtained by Berding et al. (2016), who observed an increase in gut maturation, modulated colonic and fecal microbial composition, and reduced proportions of opportunistic pathogens in piglets fed with GOS. In fact, the supplemented group was characterized by a higher abundance of Bacteroidetes in colon content, while *Klebsiella*, *Escherichia/Shigella*, *Eubacterium*, and *Roseburia* were more abundant in the control group.

#### Human milk oligosaccharides

Human milk oligosaccharides (HMOs), such as sialyllactose (SL), have demonstrated the ability to pass through the small intestine undigested, serving as the primary prebiotics for infants. Additionally, it contributes to infant health, growth, and development by exerting antimicrobial effects (Bode 2006). Comstock et al. (2017) hypothesized that HMOs would affect systemic and gastrointestinal immunity. In fact, when the HMOs (4 g/L of MR × 15 d) were administered to colostrum-deprived newborn pigs, they induced an increase in the memory effector T cells in the mesenteric lymph node cell population, indicating a general increased surveillance capacity of the intestinal immune system in HMO-fed noninfected pigs (rotavirus [RV]). In addition, Li et al. (2014) observed a reduction in diarrhea incidence when supplementing HMO and prebiotics (3.6 g + 0.4 g/L MR × 15 d) in piglets infected with RV by modulating colonic microbiota with an increase in *Lachnospiraceae* and immune response to RV infection (increase of IFN-γ, IL-10). Obelitz-Ryom et al. (2018) investigated the effects of dietary supplementation with sialyllactose (6.7 g/L MR × 19 d) in preterm piglets. Their findings demonstrated that sialyllactose had no significant impact on growth performance, intestinal morphology, gut microbiota composition, or systemic blood parameters.

The combined effect of probiotics and prebiotics was evaluated through the administration of a symbiotic product containing *Enterococcus faecium* and *Saccharomyces cerevisiae*, together with inulin, immunoglobulins, vitamins, and selenium (Girard et al. 2021). A single 2 mL dose was provided within the first 10 h after birth. This supplementation improved growth performance, as indicated by higher BW and ADG, particularly during the post-weaning period (d 39 post-weaning), and reduced the incidence of diarrhea in the treated group. The authors attributed these effects to an increased abundance of Lactobacillus in the fecal microbiota during the suckling period.

Probiotics and prebiotics are well-established feed additives that provide multiple health benefits to the host, effects that are also evident in suckling piglets. Analysis of the data indicates that these compounds, in particular probiotics, can enhance piglet growth and, specifically, modulate the intestinal microbiota by promoting the colonization of beneficial bacterial species. However, their effects on intestinal morphology remain inconclusive, although they have been shown to influence the activity of enzymes such as maltase and lactase and to increase pro-inflammatory cytokines in piglets challenged with RV. This immunomodulatory effect contributed to a reduced incidence of diarrhea, as the piglets were better able to cope with intestinal inflammation.

## Conclusion

Ensuring optimal nutrition during the suckling period is crucial for improving neonatal piglet survival and long-term growth performance. The present systematic review critically evaluated the effects of early nutritional interventions, including cross-species colostrum, MRs, and direct oral supplementation with functional additives, on survival, growth performance, and gut health-related parameters in suckling piglets. Given the limited availability of comprehensive reviews focusing specifically on nutritional strategies applied to piglets, this study provides an integrated overview of current evidence in this field.

When natural colostrum intake is insufficient, supplemental porcine colostrum is the most effective alternative, although its practical application in commercial settings remains challenging. Therefore, bovine colostrum represents a feasible substitute that can support immune maturation and survival, despite the absence of species-specific immunoglobulin transfer, while goat colostrum shows potential but requires further investigation.

MR supplementation, particularly when provided alongside sow milk, represents a practical and effective strategy to enhance growth performance, increase IGF-1 levels, and, in some cases, improve survival and reduce diarrhea incidence. However, its effects on intestinal morphology and microbiota composition remain inconsistent across studies, indicating that formulation, timing, and delivery methods require further optimization. Functional nutritional supplements offer additional opportunities to enhance piglet health. AAs, especially arginine, demonstrate consistent benefits in supporting intestinal development, immune modulation, and growth performance. Probiotics and prebiotics contribute to microbiota balance, immune maturation, and the reduction of intestinal disorders, although their efficacy varies depending on strain selection and administration protocols. In contrast, the effects of protein, vitamins, and minerals appear more context-dependent and require further targeted research.

Overall, the most effective nutritional approach for suckling piglets involves an integrated strategy that ensures adequate colostrum intake, supports milk availability through supplementation when necessary, and incorporates targeted functional additives to address specific physiological challenges. Future research should focus on standardizing supplementation protocols, identifying optimal dosing and timing strategies, and clarifying long-term effects on post-weaning performance and health.

## Supplementary Material

skag095_Supplementary_Data

## Data Availability

The authors declare that all data in the article are true and reliable and are available on demand.

## Abbreviations

A-CH50: alternative complement pathway activity
ADG: average daily gain
ATTD: apparent total tract digestibility
BW: body weight
C-CH50: classical complement pathway activity
CD: crypt depth
CD3^−^: cluster of differentiation 3
CD16^+^: cluster of differentiation 16
CLD: claudin
COX: cyclooxygenase
CP: crude protein
DHA: docosahexaenoic acid
EPA: eicosapentaenoic acid
GE: gross energy
G:F: gain to feed ratio
GPX: glutathione peroxidase
HAS: human serum albumin
Ig: immunoglobulin
Ile: ileum
IGF-1: insulin growth factor-1
IL: interleukin
Jej: jejunum
MUFA: monounsaturated fatty acids
NEFA: non-esterified fatty acids
NK: natural killer cells
NO: nitric oxide
NOS: nitric oxide synthase
OCLN: occludin
PGE2: prostaglandin
PUFA: polyunsaturated fatty acids
sIg: secretory immunoglobulin
TLR: toll-like receptor
TNF: tumor necrosis factor
VH: villous height
VH:CD: villous height to crypt depth ratio
VW: villous width
WBC: white blood cell
ZO-1: zonula occludens
LBW: low birth weight
