# Supplementary material for: A systematic review of the efficacy of complementary colostrum, milk, and oral nutritional and dietary solutions on the health, growth, and gut health of suckling piglets
Source: J Anim Sci. 2026 Apr 1;104:skag095. doi: 10.1093/jas/skag095 (PMC13293068; doi:10.1093/jas/skag095)
Supplement: skag095_Supplementary_Data [file skag095_supplementary_data.docx]

**Supplementary Table S1**. Complete list of articles included in the review.

|  | Title | Author | DOI |
| --- | --- | --- | --- |
| 1 | Docosahexaenoic acid concentrations in retinal phospholipids of piglets fed an infant formula enriched with long-chain polyunsaturated fatty acids: effects of egg phospholipids and fish oils with different ratios of eicosapentaenoic acid to docosahexaenoic acid | Alessandri et al., 1998 | 10.1093/ajcn/67.3.377 |
| 2 | Supplementation with Lactobacillus paracasei or Pediococcus pentosaceus does not prevent diarrhoea in neonatal pigs infected with Escherichia coli F18 | Andersen et al., 2017 | 10.1017/S000711451700160X |
| 3 | Short-chain fructo-oligosaccharides supplementation to suckling piglets: Assessment of pre- and post-weaning performance and gut health | Ayuso et al., 2020 | 10.1371/journal.pone.0233910 |
| 4 | Supplementing day-old pigs with bovine colostrum or milk replacer | Bandyk & Hines, 1986 | 10.4148/2378-5977.6160 |
| 5 | Prebiotics and Bioactive Milk Fractions Affect Gut Development, Microbiota, and Neurotransmitter Expression in Piglets | Berding et al., 2016 | 10.1097/MPG.0000000000001200 |
| 6 | Optimizing DHA levels in piglets by lowering the linoleic acid to alpha-linolenic acid ratio | Blank et al., 2002 | 10.1194/jlr.m200152-jlr200 |
| 7 | Milk Supplementation: Effect on piglets performance, feeding behavior and sows physiological condition during the lactation period | Chem et al., 2023 | 10.31893/jabb.23007 |
| 8 | Dietary Human Milk Oligosaccharides but Not Prebiotic Oligosaccharides Increase Circulating Natural Killer Cell and Mesenteric Lymph Node Memory T Cell Populations in Noninfected and Rotavirus-Infected Neonatal Piglets | Comstock et al., 2017 | 10.3945/jn.116.243774 |
| 9 | Effect of two milk supplements and two ways of administration on growth performance, welfare and fecal microbial ecology of suckling piglets | Correa et al., 2023 | 10.3389/fvets.2023.1050414 |
| 10 | Supplementing formula-fed piglets with a low molecular weight fraction of bovine colostrum whey results in an improved intestinal barrier | De Vos et al., 2014 | 10.2527/jas.2013-6437 |
| 11 | Effects of energy supplementation to neonatal (very) low birth weight piglets on mortality, weaning weight, daily weight gain and colostrum intake | Declerck et al., 2016 | 10.1016/j.livsci.2015.11.015 |
| 12 | Supplementation of tributyrin improves the growth and intestinal digestive and barrier functions in intrauterine growth-restricted piglets | Dong et al., 2016 | 10.1016/j.clnu.2015.03.002 |
| 13 | Extra arginine supplementation during the suckling period alleviates weaning stress through the regulation of dendritic cells and Notch2 signaling in piglets | Dong et al., 2022 | 10.1039/D1FO03720J |
| 14 | Supplemental skim milk before and after weaning improves growth performance of pigs | Dunshea et al., 1999 | 10.1071/AR98180 |
| 15 | Supplemental vitamin C and yeast cell wall beta-glucan as growth enhancers in newborn pigs and as immunomodulators after an endotoxin challenge after weaning | Eicher et al., 2006 | 10.2527/jas.2005-770 |
| 16 | Oral N-carbamylglutamate supplementation increases protein synthesis in skeletal muscle of piglets | Frank et al., 2007 | 10.1093/jn/137.2.315 |
| 17 | Effect of the administration of copper, vitamins A and D and bovine colostrum on performances, antioxidant and micronutrients status and microbiome in lactating piglets on a commercial farm | Galiot et al., 2021 | 10.1016/j.livsci.2021.104609 |
| 18 | A Single Dose of Synbiotics and Vitamins at Birth Affects Piglet Microbiota before Weaning and Modifies Post-Weaning Performance | Girard et al., 2021 | 10.3390/ani11010084 |
| 19 | Importance of neonatal immunoglobulin transfer for hippocampal development and behaviour in the newborn pig | Goncharova et al., 2017 | 10.1371/journal.pone.0180002 |
| 20 | n−3 and n−6 fatty acid enrichment by dietary fish oil and phospholipid sources in brain cortical areas and nonneural tissues of formula-fed piglets | Goustard-Langelier et al., 1999 | 10.1007/s11745-999-331-6 |
| 21 | Characterization of the Intestinal Lactobacilli Community following Galactooligosaccharides and Polydextrose Supplementation in the Neonatal Piglet | Hoeflinger et al., 2015 | 10.1371/journal.pone.0135494 |
| 22 | Dietary Long-Chain PUFA Enhance Acute Repair of Ischemia-Injured Intestine of Suckling Pigs | Jacobi et al., 2012 | 10.3945/jn.111.150995 |
| 23 | Development of Intestinal Immunoglobulin Absorption and Enzyme Activities in Neonatal Pigs Is Diet Dependent | Jensen et al., 2001 | 10.1093/jn/131.12.3259 |
| 24 | Jejunal inflammatory cytokines, barrier proteins and microbiome-metabolome responses to early supplementary feeding of Bamei suckling piglets | Jin et al., 2020 | 10.1186/s12866-020-01847-y |
| 25 | Early-Life Supplementation of Bovine Milk Osteopontin Supports Neurodevelopment and Influences Exploratory Behavior | Joung et al., 2020 | 10.3390/nu12082206 |
| 26 | Effect of live yeast Saccharomyces cerevisiae supplementation on the performance and cecum microbial profile of suckling piglets | Kiros et al., 2019 | 10.1371/journal.pone.0219557 |
| 27 | Human milk oligosaccharides shorten rotavirus-induced diarrhea and modulate piglet mucosal immunity and colonic microbiota | Li et al., 2014 | 10.1038/ismej.2014.10 |
| 28 | Natural killer cell populations and cytotoxic activity in pigs fed mother’s milk, formula, or formula supplemented with bovine lactoferrin | Liu et al., 2013 | 10.1038/pr.2013.125 |
| 29 | Early Supplementation of Phospholipids and Gangliosides Affects Brain and Cognitive Development in Neonatal Piglets | Liu et al., 2014 | 10.3945/jn.114.199828 |
| 30 | Impact of birth weight and neonatal nutritional interventions with micronutrients and bovine colostrum on the development of piglet immune response during the peri-weaning period | Lo Verso et al., 2020 | 10.1016/j.vetimm.2020.110072 |
| 31 | The effect of a single, early-life administration of a probiotic on piglet growth performance and faecal microbiota until weaning | Luise et al., 2021 | 10.1080/1828051X.2021.1952909 |
| 32 | Milk replacers supplemented with either L-arginine or L-carnitine potentially improve muscle maturation of early reared low birth weight piglets from hyperprolific sows | Madsen et al., 2018 | 10.1017/S175173111700132X |
| 33 | Efficacy of energy supplementation on growth performance and immune response of suckling pigs | Manzke et al., 2018 | 10.1093/jas/sky335 |
| 34 | Evaluation of Immunoglobulin G Absorption from Goat Colostrum by Newborn Piglets | Martinez Miro et al., 2020 | 10.3390/ani10040637 |
| 35 | Oral ingestion of colostrum alters intestinal transforming growth factor-beta receptor intensity in newborn pigs | Mei et al., 2006 | 10.1016/j.livsci.2006.06.017 |
| 36 | Supplementing Colostrum from Multiparous Sows: Effects on Performance and Health in Piglets from Gilts in Farm Conditions | Miguel et al., 2021 | 10.3390/ani11092563 |
| 37 | Effects of colostrum, and protein and energy supplementation on survival and performance of low-birth-weight piglets | Moreira et al., 2017 | 10.1016/j.livsci.2017.06.006 |
| 38 | Effect of cross-fostering and oral supplementation with colostrums on performance of newborn piglets | Muns et al., 2014 | 10.2527/jas.2013-6858 |
| 39 | Effect of oral supplementation with different energy boosters in newborn piglets on pre-weaning mortality, growth and serological levels of IGF-I and IgG | Muns et al., 2017 | 10.2527/jas.2016.0958 |
| 40 | Bovine Milk Oligosaccharides with Sialyllactose for Preterm Piglets | Obelitz-Ryom et al., 2018 | 10.3390/nu10101489 |
| 41 | Half-life of porcine antibodies absorbed from a colostrum supplement containing porcine immunoglobulins | Polo et al., 2012 | 10.2527/jas.53716 |
| 42 | Effect of artificial rearing of piglets on the volume densities of M cells in the tonsils of the soft palate and ileal Peyer’s patches | Prims et al., 2017 | 10.1016/j.vetimm.2016.12.009 |
| 43 | Intestinal Development and Fatty Acid Binding Protein Activity of Newborn Pigs Fed Colostrum or Milk | Reinhart et el., 1992 | 10.1159/000243868 |
| 44 | Alternative and classical complement pathway activity in sera from colostrum-fed and colostrum-deprived neonatal pigs. | Renshaw & Gilmore 1980 | https://www.ncbi.nlm.nih.gov/ pmc/articles/PMC1458228/ |
| 45 | Dietary bovine lactoferrin increases intestinal cell proliferation in neonatal piglets | Reznikov et al., 2014 | 10.3945/jn.114.196568 |
| 46 | Nourishing neonatal piglets with synthetic milk and Lactobacillus sp. at birth highly modifies the gut microbial communities at the post-weaning stage | Sampath et al., 2022 | 10.3389/fmicb.2022.1044256 |
| 47 | Early-Life Intervention of Lactoferrin and Probiotic in Suckling Piglets: Effects on Immunoglobulins, Intestinal Integrity, and Neonatal Mortality | Sarkar et al., 2023 | 10.1007/s12602-022-09964-y |
| 48 | Additional vitamin E required in milk replacer diets that contain canola oil | Sauer et al., 1997 | 10.1016/S0271-5317(96)00256-4 |
| 49 | A Single Dose of Fat-Based Energy Supplement to Light Birth Weight Pigs Shortly After Birth Does Not Increase Their Survival and Growth | Schmitt et al., 2019 | 10.3390/ani9050227 |
| 50 | Effects of oral glutamine supplementation on jejunal morphology, development, and amino acid profiles in male low birth weight suckling piglets | Schregel et al., 2022 | 10.1371/journal.pone.0267357 |
| 51 | Beta-lactoglobulin as a potential modulator of intestinal activity and morphology in neonatal piglets | Sutton & Alston-Mills, 2006 | 10.1002/ar.a.20327 |
| 52 | Dietary l-arginine supplementation enhances the immune status in early-weaned piglets | Tan et al., 2008 | 10.1007/s00726-008-0155-1 |
| 53 | Drenching Bovine Colostrum, Quercetin or Fructo-Oligosaccharides Has No Effect on Health or Survival of Low Birth Weight Piglets | Van Tichelen et al., 2021 | 10.3390/ani12010055 |
| 54 | The Effect of Drenching (Very) Low Birth Weight Piglets with a Dense, Concentrated Milk Replacer at Farms with Differing Farrowing Management | Van Tichelen et al., 2023 | 10.3390/ani13010063 |
| 55 | Effects of bovine colostrum on performance, survival, and immunoglobulin status of suckling piglets during the first days of life | Viehmann et al., 2015 | 10.17221/8404-CJAS |
| 56 | Performance of low birth-weight piglets upon protein-energy and/or colostrum supplementation | Viott et al., 2018 | 10.1590/1678-4162-9798 |
| 57 | Dietary supplementation with β-hydroxy-β-methylbutyrate calcium during the early postnatal period accelerates skeletal muscle fibre growth and maturity in intra-uterine growth-retarded and normal-birth-weight piglets | Wan et al., 2016 | 10.1017/S0007114516000465 |
| 58 | Calcium does not inhibit iron absorption or alter iron status in infant piglets adapted to a high calcium diet | Wauben & Atkinson, 1999 | 10.1093/jn/129.3.707 |
| 59 | Effect of Dietary Fat Level on Growth and Lipogenesis in the Colostrum Deprived Neonatal Pig | Wolfe et al., 1977 | 10.1093/jn/107.12.2100 |
| 60 | The effect of birth weight and feeding of supplemental milk replacer to piglets during lactation on preweaning and postweaning growth performance and carcass characteristics | Wolter et al., 2002 | 10.2527/2002.802301x |
| 61 | Oral administration of N-carbamylglutamate might improve growth performance and intestinal function of suckling piglets | Zeng et al., 2015 | 10.1016/j.livsci.2015.09.004 |
| 62 | 'Dietary Arginine Supplementation Affects Intestinal Function by Enhancing Antioxidant Capacity of a Nitric Oxide–Independent Pathway in Low-Birth-Weight Piglets | Zheng et al., 2018 | 10.1093/jn/nxy198 |
